# Urban heat Islands shape epiphytic communities of lichens and bryophytes

**DOI:** 10.1007/s11252-026-01930-8

**Published:** 2026-02-21

**Authors:** Tim Claerhout, Laurens B. Sparrius, Paul J.A. Keßler, Michael Stech

**Affiliations:** 1 Hortus botanicus Leiden, Leiden University, Leiden, The Netherlands; 2 Naturalis Biodiversity Center, Leiden, The Netherlands; 3 Institute of Biology Leiden, Leiden University, Leiden, The Netherlands; 4 Dutch Bryological and Lichenological Working Group, Utrecht, The Netherlands; 5 Leiden University, Leiden, The Netherlands

**Keywords:** Urban ecology, Biomonitoring, Epiphyte, Microclimate

## Abstract

Lichens and bryophytes are renowned for their use as bioindicators of environmental pollution and climate change. In urban environments, climate change may directly affect the temperature regime, thereby worsening the intensity of the urban heat island effect (UHI). Using lichens and bryophytes as bioindicators for the UHI allows urban planners and policy makers to mitigate the UHI in a targeted approach. Here, we investigated whether and how the diversity and community composition of epiphytic lichens, algae and bryophytes are influenced by the UHI gradient. We collected species and abundance data on 303 trees from the genus *Tilia* in three Dutch cities (Amsterdam, Leiden, and Rotterdam) along an UHI gradient. Additionally, abiotic data (relative humidity, temperature, and vapour-pressure deficit) were collected using sensors on 24 trees in Rotterdam. The results show that lichens have a peak in biodiversity in zones where the UHI is intermediate, whereas bryophyte biodiversity increases linearly along the gradient. Furthermore, the biodiversity of epiphytic lichens and bryophytes increases with the age of urban trees. We identified 23 indicators for different zones along the UHI gradient. Their suitability as indicators at the macroclimatic (UHI) level was supported by their microclimatic niche according to the sensor data. Our study, therefore, provides a novel, robust biological framework for developing or refining UHI monitoring in cities.

**Supplementary Information:**

The online version contains supplementary material available at 10.1007/s11252-026-01930-8.

## Introduction

The percentage of the global population living in urban areas is expected to rise to 68% in 2050 (United Nations 2025). Consequently, the expanding urbanization is connected with changing environmental conditions in terms of temperature and humidity as well as pollution (Mayer 1999; Hass et al. 2016). One result of the interplay between these environmental conditions is that cities become “heat islands” within the rural environment.

The urban heat island effect (UHI) is defined by Ulpiani (2021) as “the alteration of the thermal balance in urban spaces compared to the near rural areas”, which may result in a temperature difference up to 12 °C (e.g., Santamouris 2015). An urban heat island is characterized by increased temperatures, reduced relative humidity (RH), and changing wind and precipitation patterns (Oke 1982, 1995; Kuttler 2008). Because of this correlation with humidity, the urban heat island has also been coined the urban drought island (UDI). Some important drivers of this effect are anthropogenic emissions, urban topology (e.g., street canyons and urban connectedness), building materials with a low albedo (i.e., the fraction of light that is reflected by a body or surface), and a lower prevalence of water bodies, open soil, and vegetation. For a more conceptual understanding of the UHI, we refer to Lee et al. (2020), Ulpiani (2021) and Wang et al. (2021).

The UHI becomes especially important in conjunction with global warming. It has been shown that climate change has a synergistic influence on the UHI by increasing the number of days and nights with intense UHI episodes (Khan et al. 2023). It is expected that this synergistic effect will increase the number of heat waves, with negative effects on the health of city dwellers (van Hove et al. 2011; Steeneveld et al. 2011), and also on the non-human species communities of the urban ecosystems.

Knowing how different species react to changes in the urban environment allows us to properly design urban (green) spaces to counter climate-related threats to biodiversity (Wilby and Perry 2006) and provide ecosystem services that can alleviate urban problems, such as increased temperatures and pollution (Seddon et al. 2020). For example, a detailed understanding of the different tree species that can thrive in urban environments is essential for a more efficient urban climate regulation (Lanza and Stone 2016). In addition, identifying species that are indicative of specific urban environmental conditions allows the development of a simple, low-cost biomonitoring protocol that complements existing monitoring regimes based on physical measurements of temperature, concentration of pollutants and others.

Two such groups of bioindicators are lichens and bryophytes. Historically, epiphytic lichens and bryophytes have mainly been used with regard to monitoring air pollution ever since the mid-19th century (Nylander 1866; Skye 1979; Tessier and Boisvert 1999; Sparrius 2007; Gerdol et al. 2014; Anderson et al. 2022). Most studies published between 2011 and 2020 using lichens as bioindicators targeted urban and industrial areas (Abas 2021), with a clear focus on the effect of air pollution. However, their applicability is not restricted to air pollution, as proven by, e.g., van Herk et al. (2002) and Aptroot et al. (2021), but can be used also to indicate the effects of climate change.

Research regarding the effect of climate change and the UHI has received an increase in attention over the last 10 years. Several studies have found functional traits of lichens which may serve as ecological indicators of climate change (Matos 2016) and the UHI. Matos et al. (2015) found that, in a Mediterranean dryland ecosystem, lichens with a *Trentepohlia* photobiont and fruticose lichen species are negatively correlated with aridity, while lichens with a cyanobacterium as photobiont (i.e., cyanolichens) and crustose, squamulose, and foliose lichen species are positively correlated. Koch et al. (2019) proposed that lichens with a *Trentepohlia* photobiont, cyanolichens, crustose lichens, and lichens with isidia are indicative of low urbanization, while lichens with a chlorococcoid photobiont, foliose lichens, and lichens with soredia and pruinose thalli are indicative of medium/high urbanization. Munzi et al. (2014) studied lichen functional groups and noticed a correlation between hygrophytic lichens and the decrease in humidity in urban environments. In terms of taxonomic diversity indices, diversity as a whole was positively correlated with annual mean temperature and relative humidity (RH) in Matos et al. (2017). Most recently, Claerhout et al. (2025) investigated the specific thallus mass (STM; dry thallus mass per unit of area) of urban lichens and found that STM increased significantly along a gradient of the UHI, indicating that the UHI affects not only the species community, but also the physiology of individual lichens. Lichen indicators for monitoring climate change have already been proposed and applied in Germany by Stapper and John (2015) and the association of German engineers (VDI; Verein Deutscher Ingenieure 2017, 2023), and in other European countries by Counoy et al. (2025).

Regarding the influence of urbanization on epiphytic bryophytes, Żołnierz et al. (2022) found that the bryophyte diversity in the Polish city of Wrocław showed a negative trend with variables related to increased urbanization, such as a lower NDVI (normalized difference vegetation index) and a higher UHI, among other factors influencing species diversity. Oishi and Hiura (2017) found no trend in species diversity along an urbanization gradient in Tokyo, but did see a significant increase in epiphytic bryophyte coverage and a decrease of hygrophilous bryophytes with increased urbanization (and as an extension, the relative humidity). The UHI was found to have an influence on the bryophyte composition up to 1000 m from the sampling site (Oishi 2019). However, no bryophyte indicator species have been identified in relation to the UHI.

Studies on the ecology of epiphytic lichens and bryophytes in urban environments mainly consider large-scale factors such as city-wide pollution and the UHI, as well as phorophyte characteristics (e.g. tree species, tree height, bark texture, bark chemistry). For the Netherlands, most recently Timans et al. (2025) disentangled the interplay of environmental parameters and phorophyte characteristics shaping lichen communities on street trees in Amsterdam. In this paper, species richness declined with sun exposure, decreasing humidity, and extreme shading, but increased with the water-holding capacity and the roughness of bark. However, scientific papers using microclimatic data from urban trees to explain epiphyte biodiversity are difficult to find since macroclimatic data is more readily available, and microclimatic data requires a higher time investment to collect. Therefore, we lack a fundamental understanding of the concordance between macroclimatic factors such as the UHI and microclimatic variables such as temperature, relative humidity and vapour-pressure deficit (VPD) on the scale of the epiphyte itself.

In this paper, we aim to (1) investigate the patterns of diversity and community composition of epiphytic lichens and bryophytes on street trees along an urban heat island (UHI) gradient in three cities in the Netherlands (Amsterdam, Leiden and Rotterdam); (2) discern the relative influence of tree-related and environmental macro- and microclimatic variables in explaining these patterns; and (3) identify indicators for the UHI and calculate their microclimatic niche using sensor data.

## Materials and methods

### Study sites and plot selection

This study was carried out in three cities in the Netherlands: Amsterdam (52.36°N, 4.90°E), Leiden (52.16°N, 4.49°E), and Rotterdam (51.92°N, 4.47°E). These cities have an average annual temperature of 10.7 °C, 10.8 °C and 10.8 °C, and an average annual precipitation of 844 mm, 940 mm, and 835 mm, respectively (2024; climate-data.org). As of January 1st, 2025, Leiden was the smallest city with 130,613 inhabitants, followed by Rotterdam with 672,330 and Amsterdam with 935,793 inhabitants (overheid.nl). Together, the three cities comprised about 10% if the total population of the Netherlands (18,045,532; overheid.nl). Nitrogen levels are generally still high in the Netherlands, although a decreasing trend in nitrogen pollution (from 2700 mol N/ha in 1990 to 1315 mol N/ha in 2023; Mijnen-Visser et al. 2025) and particulate matter (PM; Berkhout et al. 2024) is visible.

**Fig. 1:**
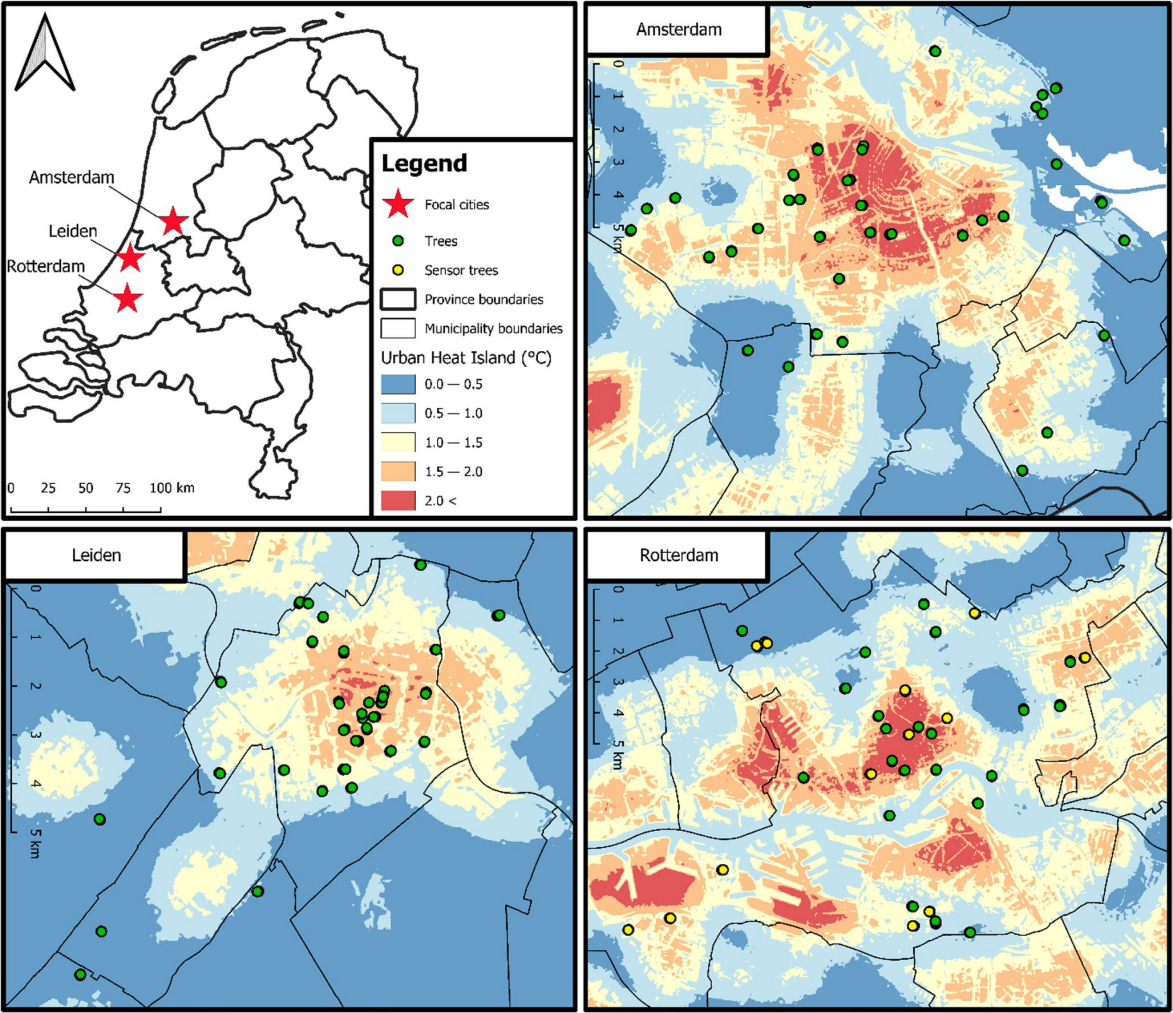
Locations of the sampling sites across the three focal cities: Amsterdam, Leiden and Rotterdam. Each sampling site (green and yellow dots) comprised three *Tilia* trees within a 100 m^2^ grid square. Yellow sampling sites are those where sensors (temperature and relative humidity) were placed on the trees. The background layer depicts the five urban heat island zones across which sampling sites were randomly selected

Suitable sampling sites were selected by plotting a grid of 100 m^2^ squares on the three cities in QGIS v.3.34. The number of x by y-squares and the top-right coordinates were (1) for Amsterdam: 299 × 266, 52.426691, 4.755983; (2) for Leiden: 97 × 93, 52.184968, 4.441897; and (3) for Rotterdam: 527 × 284, 51.994959, 4.115199. Based on the tree management plans from the municipalities of Amsterdam, Leiden and Rotterdam, only squares with three or more trees from the genus *Tilia* were kept. This genus was chosen as it is readily available as an urban tree species in all three focal cities. Suitable squares with *Tilia* trees were selected per city across five UHI zones (dark blue (DB): 0–0.5 °C; blue (B): 0.5–1.0 °C; yellow (Y): 1.0–1.5 °C; orange (O): 1.5–2.0 °C; red (R): > 2.0 °C). The UHI zones resulted from the UHI map by Remme et al. (2017), which was based on average windspeed (at 10 m above ground level) and population density (total population in a radius of 10 km), recalibrated to account for soil sealing (% within a 1 km radius), vegetation cover (30 m radius) and land cover classes (30 m radius; CBS 2017). For this paper, the UHI map was modified by classifying the continuous UHI data into five UHI zones (Fig. 1).

Seven squares were selected per UHI zone per city, except for the UHI zones “DB” and “R” in Leiden, for which only 5 squares each met the imposed criteria. Final grid square selection followed a stratified sampling procedure, where squares were selected randomly within each UHI zone. The selected squares (*N* = 101) served as the sampling sites of the subsequent field work. However, when a site was deemed unsuitable during field work, an adjacent square was selected, as stipulated in Asta et al. (2002), but modified by sampling back-up trees initially in the same street when there was no opportunity to sample three trees in the same plot. If the sampling site was unsuitable, the next square to be chosen was the most northern one. If the same problem occurred, square selection continued in a clockwise manner.

### Tree selection and environmental data 

In total, 303 trees (i.e., 3 per grid square) were sampled across the three cities, all with a diameter at breast height (DBH) of 30–80 cm, to reduce the impact of bark texture of very young or very old trees, and a maximum inclination of 10°. Furthermore, additional information such as the tree species, the DBH, and the coordinates was collected. To account for the effect of heat radiation from the ground upwards and from above as well as the reduced relative humidity (RH), the “Degree of Concealment” (DoC; estimate of the percentage of stony surface (incl. pavement and gravel) in a 5 m radius around the tree) and light availability (LiAv; estimate of the percentage of open air when looking south) was estimated *in situ.* To account for influences related to traffic and pollution, we extracted the following macro-environmental data from RIVM (de Smet et al. 2023): (1) “traffic sound intensity” as a proxy for traffic-related pollution (2) Nitrogen dioxide (NO_2_; concentration of nitrogen dioxide (µg/m^3^) in 2021), (3) PM_2.5_ (concentration of particulate matter with a diameter  < 2.5 μm (µg/m^3^) in 2021) and (4) PM_10_ (concentration of particulate matter with a diameter  < 10 μm (µg/m^3^) in 2021). All macro-environmental data sources had a spatial resolution of 20 m. No environmental data relating to ammonia pollution was included since ammonia concentrations are relatively low in the study area compared to the more rural areas in the Netherlands where agriculture is prevalent. Since no gradient can be established, including it would make the variable redundant.

Local temperature and RH data were collected using iButton^®^ DS1923-F5# Hygrochron data loggers to quantify the UHI across different urban topologies and to identify microclimatic differences. Data loggers were placed on 26 trees at 13 sites in Rotterdam (Fig. 1), which were dispersed along the UHI gradient and had a maximum number of indicators from the indicator species analysis (ISA) present (see section “Data analyses”). Loggers were placed on the north and south side of two trees per site at a height of 3 m above the ground. To minimize the effect of direct solar radiation and reduce temperature bias, the loggers were encased by 10 cm long pipe wrap isolation tubes where the top opening was closed with one layer of insulation tape and duct tape each to keep them insulated and waterproof (Terando et al. 2017). Sensors gathered climate data in two seasons: 44 days in summer, divided over three sampling periods, and 29 days in winter, divided over two sampling periods (Online Resource 7). Sensor data were analysed using the R-package “myClim” (Man et al. 2023). From the temperature and RH data, two other variables were calculated, namely, FDD (freezing degree days; # days where T < 0 °C) and VPD (vapour-pressure deficit; kPa). The VPD measures the difference between the amount of moisture in the air and the amount of moisture the air could hold when it is fully saturated. Because it resembles the air’s drying capacity, it is a more ecologically meaningful variable in our study than RH. An R-script was made whereby any combination of sensors could be plotted against each other in line plots and/or raster graphs. Furthermore, an NMDS analysis was performed on the vegetation data of every tree with the sensor data of that respective tree (T, RH, FDD, VPD). This sensor data was first aggregated into seasons (summer (suffix “_S”) and winter (suffix “_W”)) and trimmed to account for correlation between variables (variables with |*ρ*| > 0.7 were excluded). This resulted in the final set of variables “RH_S”, “RH_W”, “T_S”, “T_W”, “VPD_S” and “VPD_W” (where the suffixes “_S” and “_W” refer to the summer and winter sampling periods, respectively). Finally, mean values of each variable were calculated for every species present on the sensor trees.

### Vegetation survey

To quantify the bryophyte and lichen composition on each tree, we followed a modified procedure of Asta et al. (2002). We attached four sampling ladders (each having five 10 × 10 cm contiguous squares) to the trunk at the cardinal points (N, E, S, W), so that the upper edge of the ladder was situated 1.5 m above the highest point of the ground. A maximal shift of 20° was allowed for individual ladders to avoid parts of the trunk that were unsuitable for sampling. All bryophyte and lichen species, as well as their abundance (in percentages) were recorded within each 10 × 10 cm square. Lichens and bryophytes were identified using morphological and chemical characters in situ. Unknown species were collected and further analysed in the laboratory. Species names follow van Herk et al. (2022) for lichens and Siebel and During (2006) for bryophytes. The alga *Apatococcus ammoniophilus* Søchting, Friedl & Moestrup (commonly referred to in the literature as *Klebsormidium crenulatum* (Kützing) Lokhorst) and the total species coverage was also recorded. *Apatococcus ammoniophilus* was included since it was found to be significantly correlated with urbanization by Stapper (2022).

### Data analyses

Unless specified otherwise, statistical analyses were run using the data where species abundance was averaged over the entire sampling site, i.e. averaged over the five sampling squares of each cardinal direction, over all cardinal directions, and over the three sampled trees in each site (hereafter referred to as “averaged data”). All statistical analyses were run in R v.4.3.2 (R Core Team 2026).

Firstly, the epiphytic diversity was compiled and characterized based on ecological indicator values (EIVs or Ellenberg values: F, moisture; L, light; R, pH; N, nutrient availability; T, temperature; K, continentality; each ranging from 1 to 9) obtained from Sparrius et al. (2015) for lichens and Siebel (2005) for bryophytes. Bar graphs of the total abundance (sum of all percentages of all cardinal directions and sites) of the top 10, top 25 and of all species were made.

Secondly, we calculated multiple alpha biodiversity indices to analyse differences and trends regarding biodiversity patterns: Hill indices (*α* = 0, 0.25, 0.5, 1, 2, 4, 6, 8 and ∞) and Pielou’s evenness index J’ for every site and UHI zone level using the R packages “HillR” (Li 2018) and “BiodiversityR” (Kindt and Coe 2005), and “vegan” (Oksanen et al. 2025), respectively. Since more weight is given to abundance of species with increasing Hill indices, all Hill indices were calculated for every UHI zone after rarefaction. Hill index 1 (in essence the Shannon-Wiener index) and Pielou’s evenness index were plotted against the UHI.

Thirdly, we calculated the zeta diversity for every site as an alternative to beta diversity. This was opted for since beta diversity is insensitive to occupancy changes in common species (McGlinn and Hurlbert 2012; Hui and McGeoch 2014). Zeta diversity (ζ_i_) constitutes “the number of species shared by a given number of sites”, as defined by McGeoch et al. (2019). It provides a measure of species turnover for the combination of a given number of sites. This combination of a given number of sites equals the zeta order. The expected and observed zeta diversity decline was calculated with a confidence level set to 0.95, using the function “Zeta.decline.mc” from the R package “zetadiv” (Latombe et al. 2018). We calculated zeta diversity using presence-absence data per plot. Similarly, a Sørensen-equivalent zeta diversity decline was calculated for each city, all cities combined, and for each UHI zone. Sørensen normalisation divides the zeta values for each sample by the average number of species per site. These analyses include the following graphs: zeta diversity decline (shared percentage of species between sites in relation to the number of sites compared), the retention rate (zeta ratio in relation to the number of sites compared, indicating the probability of retaining (or discovering) a species of the same order of commonness in additional cases, as defined by (McGeoch et al. 2019), and two functions that fit the data (exponential or power-law regression fit), indicating the shape of the decline. This tells us if the community is (non-)randomly structured. For a full overview of the applications of the zeta diversity decline and retention rate, we refer to McGeoch et al. (2019).

Fourthly, we performed an NMDS-analysis with a Bray-Curtis dissimilarity matrix to visualize the community composition across the environmental variables with an emphasis on the UHI zones. Autocorrelation was tested between variables of interest by calculating the Pearson correlation coefficient *ρ*. Variables with a |*ρ*| > 0.7 were excluded from the analysis whereby retaining the ecologically most relevant variable. The final set of variables for the NMDS was: “diameter at breast height” (DBH), “degree of concealment” (DoC), “light availability” (LiAv), “traffic sound intensity”, “nitrogen dioxide“, and “urban heat island effect” (UHI). Variables related to particulate matter (PM_2.5_ and PM_10_) were excluded from the analysis.

Fifthly, we fitted generalized linear mixed models (GLMMs) on the Shannon diversity (Hill_1_) of the total dataset to identify significant factors influencing the observed biodiversity patterns. Normality was inspected and confirmed visually using QQplots and quantitatively with the Shapiro-Wilk test. Homoscedasticity was confirmed using the “allEffects”- and “plotResiduals”-function of “glmmTMB” (McGillycuddy et al. 2025). The final set of variables is equal to that of the NMDS analysis. Heteroscedasticity of the variable “LiAv” was corrected by centering and scaling the variable. Furthermore, a GLMM was fitted for lichens and bryophytes separately.

Lastly, an indicator species analysis (ISA; Dufrêne and Legendre 1997) was run to identify species that are significantly correlated with the UHI and can be used as indicators for biomonitoring the UHI in urban environments. The analysis was run using the R package “indicspecies” (De Cáceres and Legendre 2009). The “multipatt” function was run with 9999 permutations, “max.order = 2”, and adjusted p-values using the Benjamini-Hochberg procedure (Benjamini and Hochberg 1995). The final selection of indicators was obtained by first filtering species and species-combinations with a significant adjusted p-value, with component ‘A’ > 0.6 (‘A’ being the specificity, i.e., the probability that the surveyed site belongs to the target site group given the fact that the species has been found) and with component ‘B’ > 0.3 (‘B’ being the fidelity, i.e., the sample estimate of the probability of finding the species in sites belonging to the site group). These are defined by (Dufrêne and Legendre 1997). Species combinations were only included when they were not the byproduct of a combination of other indicators (e.g., species X + Y as an indicator for zone “Y” while X is an indicator of “B” and Y is an indicator of “O”) or when one of the two suggested species was indicative for another UHI zone.

## Results

### Biodiversity patterns and community composition in relation to the urban heat island

In total, 107 species of epiphytic lichens and bryophytes were found on the 303 studied *Tilia* trees (Online Resource 1 and 2). Species diversity was comparable between all three cities (87 species in Amsterdam and Rotterdam, and 90 in Leiden). Lichens were generally more diverse and common than bryophytes, and made up nine out of the ten most abundant species, together with the green alga *Apatococcus ammoniophilus*, which was also found commonly in all three cities (Online Resource 3).

Seven out of the ten most abundant species are nitrophytic species with an ecological indicator value (EIV) of N > 7 (Online Resource 2). Of all species for which EIVs were available, 88%, 73% and 89% had an EIV > 5 for EIVs L, N and T, respectively; 84%, 36% and 27% had an EIV > 6; 78%, 32% and 19% had an EIV > 7. Regarding the moisture EIV (F), 82% had a value ≤ 3, 18% of ≤ 2 and 13% of 1. These results indicate that the epiphytic communities on the sampled *Tilia* trees were generally xerophytic, photophilic, and mildly to very nitrophytic.

In general, the south and west side of the tree had a higher coverage of epiphytes, except for few species that were clearly dominant on the north and east sides. Among the top 10 most abundant species, these were *Apatococcus ammoniophilus* and *Lecanora expallens* Ach., and among the top 25 species, *Lepraria incana* (L.) Ach. as an additional species (Online Resource 3).

**Fig. 2:**
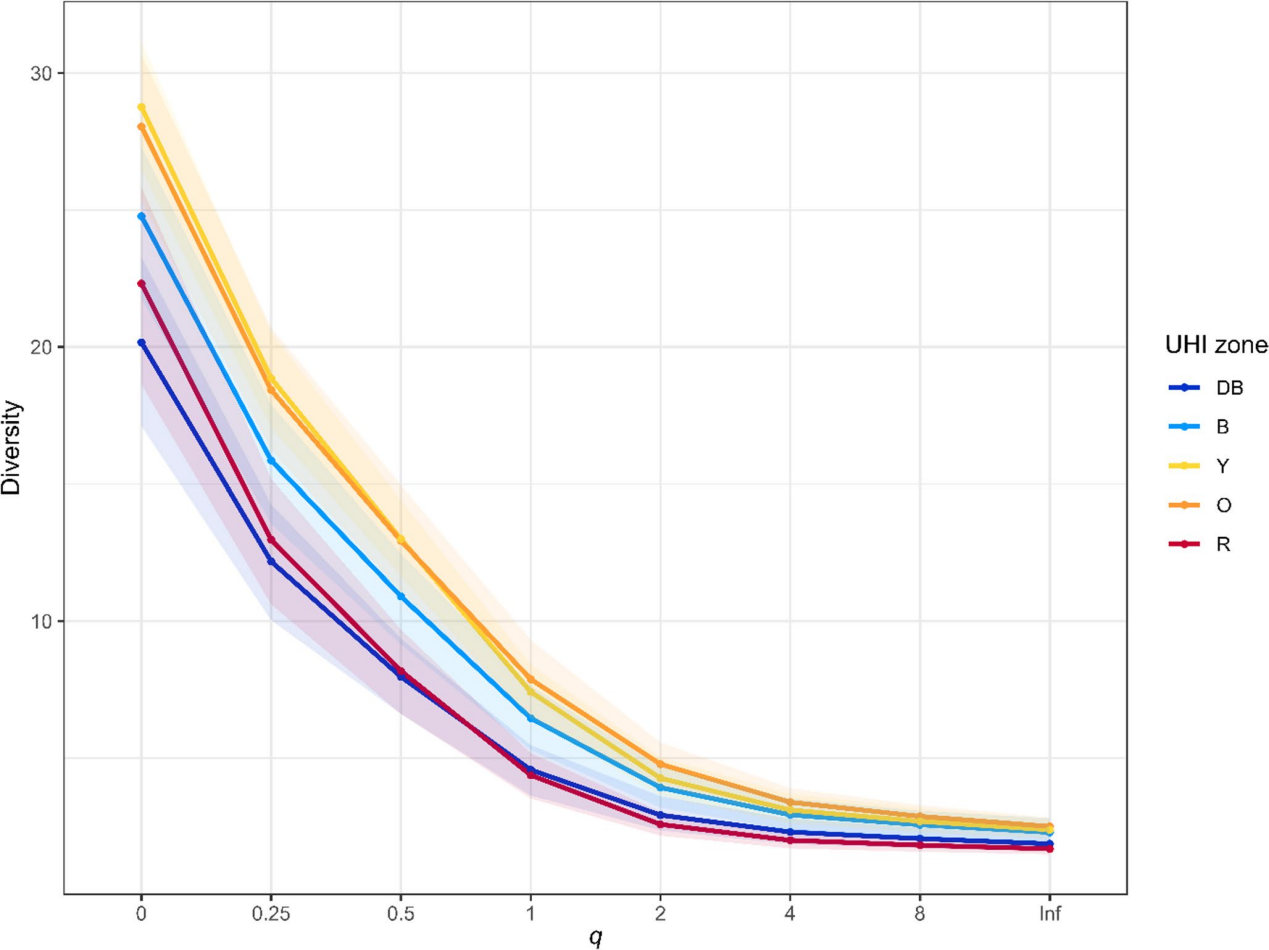
Hill index values (with 2.5% and 97.5% quantiles) across Hill indices (*q*) 0, 0.25, 0.5, 1, 2, 4, 6 and ∞ for every urban heat island zone (dark blue (“DB”): 0–0.5 °C; blue (“B”): 0.5–1.0 °C; yellow (“Y”): 1.0–1.5 °C; orange (“O”): 1.5–2.0 °C; red (“R”): > 2.0 °C)

The zones with the highest (“R”) and lowest (“DB”) urban heat island effect consistently exhibited the lowest epiphyte diversity across all Hill indices (Fig. 2; raw data of indices per site in Online Resource 4). All Hill indices exhibited the same trend in biodiversity for every UHI zone, i.e. the difference between UHI zones were independent of the weight given to species abundance.

**Fig. 3:**
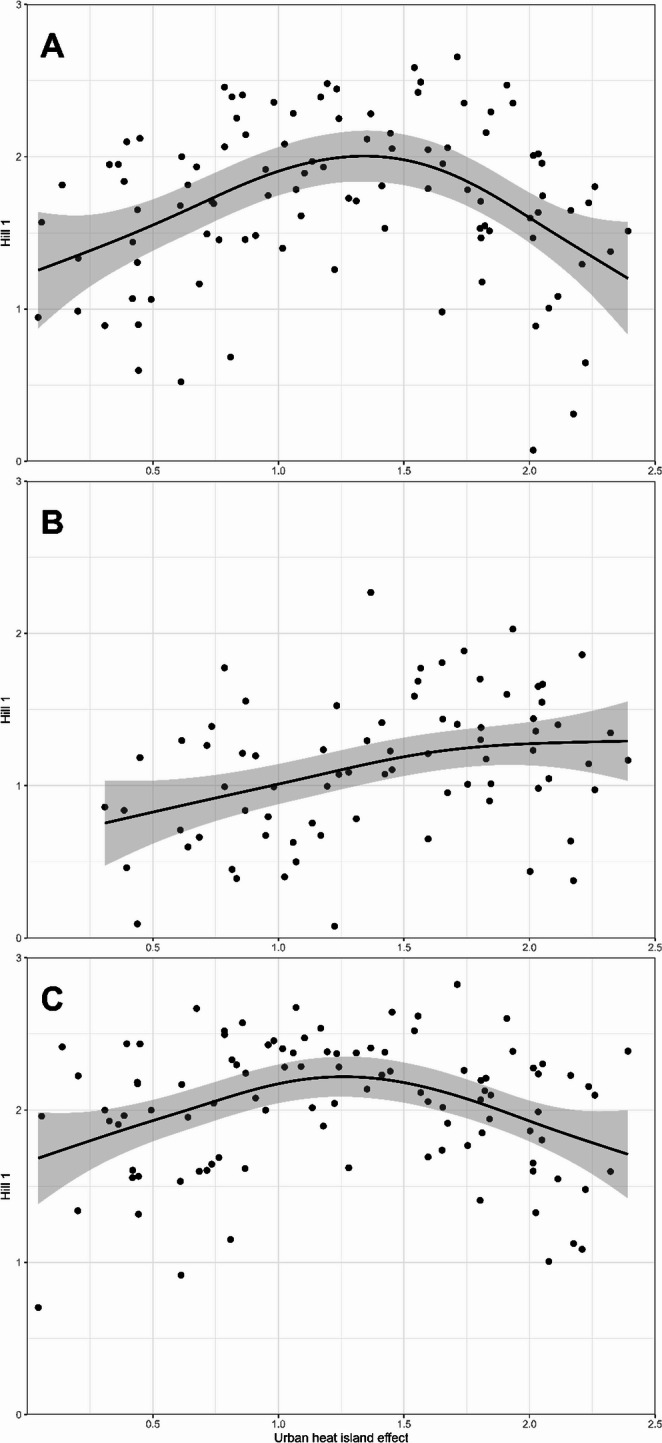
Hill index 1 across the urban heat island effect (UHI). Every point represents one sampling site. In black, a GAM (generalized additive model) is plotted. **(A)** Unimodal response of lichen and bryophyte data to the UHI. **(B)** Positive linear response of bryophyte data to the UHI. **(C)** Unimodal response of lichen data to the UHI

When plotting Hill index 1 of the entire dataset (lichens, algae, and bryophytes together) against the UHI gradient (Fig. 3A), a unimodal response between the diversity and the UHI was visible. This implies that in high and low UHI zones, the biodiversity was relatively low, compared to areas with moderate UHI. This pattern was mainly caused by the lichen diversity (Fig. 3C), while the bryophyte diversity alone showed a positive linear response to the UHI (Fig. 3B). A similar unimodal response between Pielou’s evenness index J’ and the UHI was found (Online Resource 4). Sampling sites in the medium UHI zones have a higher evenness and thus, species are more equally present, whereas the lower and higher UHI zones are more dominated by a low number of dominant species.

Zeta diversity declines rapidly and converges to ζ_i_ = 1, corresponding to the number of species shared between all sampling sites (Fig. 1A in Online Resource 5). The rapid decline illustrates the swift and almost complete turnover in species at around 30 sites. There is a small subset of widespread species that is present in all three cities. According to the retention rate (Fig. 1B in Online Resource 5), species were lost quickly across the first 20 sites, indicating the loss of rare species. After that, the graph starts to approach an asymptote at around zeta order 40, although the curve does not reach a plateau. Nonetheless, this is a good cut-off point to indicate whether species are more common or rarer. The AIC values from the last two graphs show that the parametric shape of the zeta decline fits the power law relationship best, indicating that the sampled epiphytic communities are indeed structured in some way (Fig. 1C & D in Online Resource 5).

A normalized Sørensen-equivalent of these graphs was made for every UHI zone (Fig. 2 in Online Resource 5). In the zeta diversity decline, the UHI zone “DB” consistently approached the lowest zeta diversity, while “Y” consistently had the highest zeta diversity. This is concurrent with our findings in Fig. 3. In every UHI zone except “DB”, the retention rate completely reached an asymptote. In “DB”, the retention rate dropped further at around zeta order ten and reaches zero at zeta order 16, indicating a complete species turnover in species. All zones show a better fit with the power law relationship as proven by the lower AIC values, except for “DB” with a better fit with the exponential relationship.

### Environmental parameters influencing epiphyte communities

**Fig. 4:**
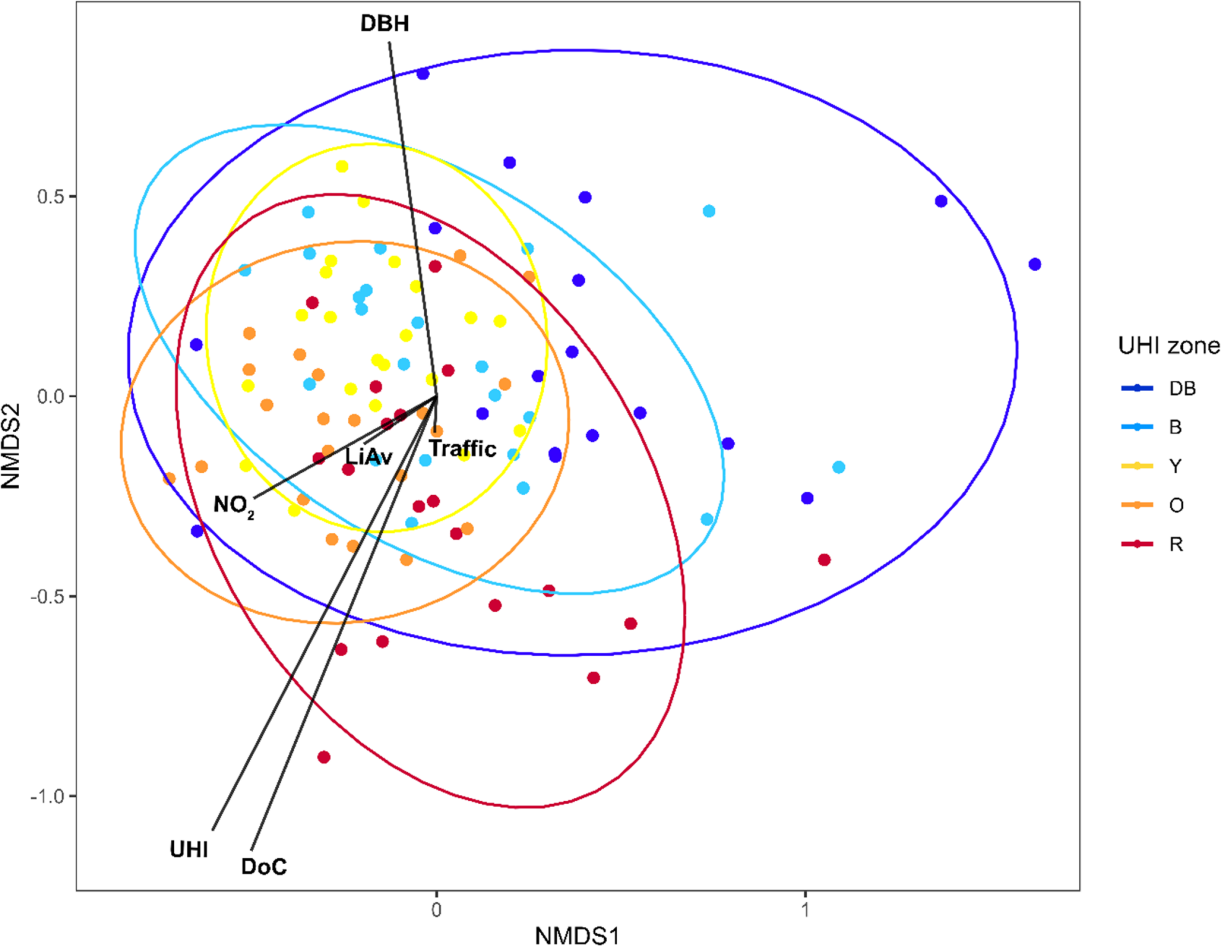
Non-metric multidimensional scaling (NMDS) plot for every urban heat island (UHI) zone (dark blue (“DB”): 0–0.5 °C; blue (“B”): 0.5–1.0 °C; yellow (“Y”): 1.0–1.5 °C; orange (“O”): 1.5–2.0 °C; red (“R”): > 2.0 °C). Points represent sites and arrows indicate the strength of the environmental gradient for the variables “DBH” (tree diameter at breast height; R^2^ = 0.17, *p* < 0.01), “DoC” (degree of concealment; R^2^ = 0.33, *p* < 0.01), “LiAv” (light availability; R^2^ = 0.011, p = n.s.), “NO_2_” (nitrogen dioxide; R^2^ = 0.07, *p* < 0.05), “Traffic” (traffic sound intensity; R^2^ = 0.002, p = n.s.) and “UHI” (R^2^ = 0.34, *p* < 0.01). Ellipses are added to indicate the clustering of sites from the same UHI zone, which share the same colour. (Final stress = 0.20, stress-based R^2^ = 0.96, fit-based R^2^ = 0.85)

The NMDS analysis of the averaged data with UHI as a grouping factor resulted in Fig. 4. The lowest UHI zone (“DB”) is partially separated from all other zones (“B”, “Y”, “O” and “R”) along the x-axis (NMDS1), while the highest UHI zone (“R”) is partially separated along the y-axis (NMDS2). This urbanization gradient corresponded to the gradient of nitrogen dioxide. Along the y-axis (NMDS2), sites are spread out along the “UHI”, “DoC” and “DBH” gradients. “Traffic intensity” and “LiAv” show a very weak effect on the spread of the sampling sites.

Moreover, a generalized linear mixed model (GLMM) was built to infer the respective influences of each environmental variable. The model was obtained by including an interaction between the polynomial of “UHI” and “LiAv”, since the amount of light influences the UHI. “DoC” was removed from the model because of a singular fit with the Shannon diversity data. “NO_2_“ and “Traffic intensity” were removed from the model because they did not have any significant influence on the diversity and because they were borderline correlated with each other and other variables. The final model for all data was: $\:{Hill\:}_{1}\:\sim\:{\left(UHI\right)}^{2}+\:{\left(UHI\right)}^{2}:LiAv+DBH$. The summary statistics for all three GLMMs can be found in Table 1.

**Table 1:** Summary statistics of the regression analysis from the original model for all data, lichens only and bryophytes only. **SE.** Standard Error. P-values indicated by “ns” (non-significant), “*” (*p* < 0.05) or “**” (*p* < 0.01). Adj. R^2^: 0.21, 0.18 and 0.19, respectively

Factor	All data			Lichens			Bryophytes		
	Estimate	SE	*p*	Estimate	SE	*p*	Estimate	SE	*p*
(Intercept)	5.79	1.68	**	2.03	0.04	**	1.14	0.05	**
UHI	3.61	3.25	ns	-0.28	0.69	ns	2.39	0.88	**
UHI^2^	-14.45	3.28	**	-2.50	0.65	**	-1.72	0.88	*
UHI: LiAv	-1.01	3.14	ns	0.01	0.01	ns	-0.02	0.02	ns
UHI^2^:LiAv	4.17	2.93	ns	0.02	0.01	ns	0.03	0.02	ns
DBH	0.09	0.04	*						

**Fig. 5:**
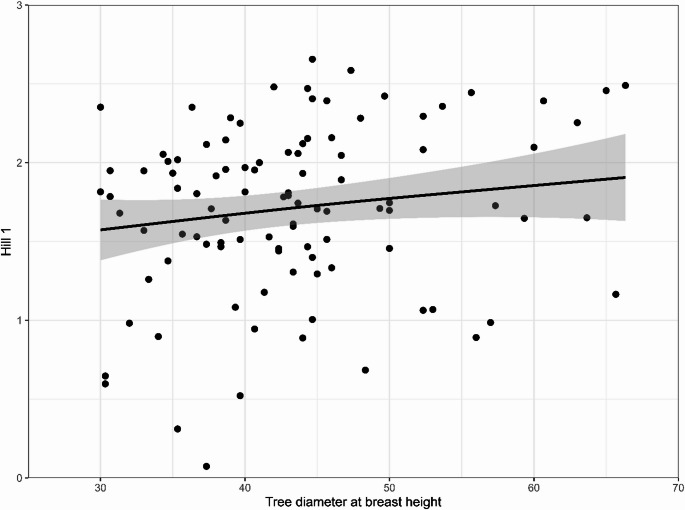
Scatterplot of diversity (Hill 1) with increasing diameter at breast height (DBH)

The second polynomial of “UHI” had a significant effect on the biodiversity of all epiphytes and lichens separately, indicating that diversity increases to a maximum at intermediate UHI and then decreases again. For bryophytes only, diversity increased linearly with the UHI, but seemed to decline at higher UHI values, explaining the borderline significant second polynomial of the bryophyte GLMM. “DBH”, as a proxy of the age of a tree, was also a significant factor in explaining the diversity of all species, but not for lichens and bryophytes separately. An increase in DBH led to a significant increase in diversity, as visualised in Fig. 5. “LiAv”, whilst not significant, was important to retain in the model because of its influence on both the temperature regime of the epiphytes and as an extension, the epiphyte composition itself.

### Indicator species analysis for the urban heat island gradient

Based on the raw data in Online Resource 6, the Indicator Species Analysis (ISA) resulted in a final set of 23 indicators across the five UHI zones. These included 17 single species (13 lichens and four bryophytes), four couples of lichen and bryophyte species, the alga *Apatococcus ammoniophilus*, and the total species coverage itself (Table 2). Two of the lichen species were included despite insignificant p-values: *Arthonia radiata* (Pers.) Ach. and *Hypotrachyna revoluta* (Flörke) Hale. *Arthonia radiata* showed high “A” and “B” values and was abundant outside of cities, but occurred mainly on younger trees with smooth bark and was often not sampled because it fell outside of the boundaries of the sampling ladder (pers. obs.). Furthermore, it shares the same photobiont (*Trentepohlia*) as the other indicators of the zone “DB”, a photobiont which is present in species that grow in more humid habitats. *Hypotrachyna revoluta* grows in semi-urban areas following personal observations (UHI zones “B” and “Y”) and showed high “A” and “B” values. Therefore, its borderline insignificant p-value should not reject the species as an indicator. On the contrary, *Lecidella elaeochroma* (Ach.) M. was rejected as an indicator despite its significant p-value, since it is a species which grows predominantly on smooth bark and its distribution is thus mainly influenced by the occurrence of young(er) trees. Finally, the total coverage of lichens and bryophytes was lower in zones “DB” and “R” and may therefore be considered as an indicator for both ends of the UHI gradient, despite its specificity (“A”) being lower than 0.6. Indicators for the lower UHI zones are crustose lichen species with a *Trentepohlia* photobiont or fruticose lichen species. Those in the intermediate UHI zones are foliose lichen species and those in the higher UHI zones are generally acrocarpous bryophytes with leaves containing hair-points that curl or fold up in some way when dry.

### Correlation of microclimatic data with the urban heat island and its indicators

In summer, the minimal and mean temperature and vapour-pressure deficit (VPD) was rising with increasing UHI, while the range of temperatures as well as the minimal, maximal and mean relative humidity (RH) decreased with increasing UHI (Online Resource 7). During winter, the same pattern was observed, except for a very slight decrease in the maximal and mean RH. Thus, no distinction could be made between the influence of temperature (UHI) and RH (the urban drought island; UDI). When arranging the seasonal means of every microclimatic variable for every indicator present on the sensor trees (total of 19 indicators, only *Arthonia radiata* and *A. spadicea* (Leighton) Frisch, Ertz, Coppins & P.F. Cannon. are missing from Table 2) along a gradient of increasing UHI zone, we saw that every temperature-related microclimate variable increased with UHI zone (Online Resource 7). Only the RH microclimate variables in summer decreased with UHI zone, except for “RH_range_S”, which increased. Similarly, all VPD microclimate variables in summer increased with the UHI, including “VPD_range_S”. No pattern is visible for RH and VPD microclimate variables in winter. An R-script to get line plots and/or raster graphs for every combination of sensors can be found in Online Resource 8.

**Table 2:** List of indicators (including species combinations) for every UHI zone (dark blue (“DB”): 0–0.5 °C; blue (“B”): 0.5–1.0 °C; yellow (“Y”): 1.0–1.5 °C; orange (“O”): 1.5–2.0 °C; red (“R”): > 2.0 °C). 0’s and 1’s indicate the potential as an indicator for the respective UHI zone. Species with superscript “1” were kept as an indicator despite insignificant p-values. Species with a superscript “2” were excluded as an indicator, despite a significant p-value. P-values are adjusted using a Benjamini-Hochberg procedure (Benjamini and Hochberg 1995). **(A)** the specificity, i.e., the probability that the surveyed site belongs to the target site group given the fact that the species has been found (Dufrêne and legendre 1997; de Cáceres and legendre 2009). **(B)** the fidelity, i.e., the sample estimate of the probability of finding the species in sites belonging to the site group (Dufrêne and legendre 1997; de Cáceres and legendre 2009)

Species	UHI zone					*p*-value	A	B
	DB	B	Y	O	*R*		Specificity	Fidelity
Total species coverage	1	0	0	0	1	0,004	0,467	1
*Anisomeridium polypori*	1	0	0	0	0	0,011	0,969	0,333
*Arthonia spadicea*	1	0	0	0	0	0,013	1	0,389
*Pseudoschismatomma rufescens*	1	0	0	0	0	0,030	0,948	0,333
*Arthonia radiata* ^1^	1	1	0	0	0	0,097	0,955	0,444
*Lecidella elaeochroma* ^2^	1	1	0	0	0	0,011	0,789	0,773
*Evernia prunastri* & *Flavoparmelia soredians*	0	1	1	0	0	0,033	0,961	0,429
*Evernia prunastri*	0	1	1	0	0	0,004	0,896	0,714
*Flavoparmelia caperata*	0	1	1	0	0	0,004	0,935	0,524
*Flavoparmelia caperata* & *F. soredians*	0	1	1	0	0	0,033	0,958	0,476
*Hypotrachyna revoluta* ^1^	0	1	1	0	0	0,055	0,833	0,667
*Parmelia sulcata*	0	1	1	0	0	0,014	0,707	0,952
*Flavoparmelia caperata* & *Lecanora chlarotera*	0	0	1	0	0	0,033	0,935	0,524
*Lecanora expallens* & *Orthotrichum affine*	0	0	1	1	0	0,033	0,914	0,476
*Physconia grisea*	0	0	1	1	0	0,004	0,680	0,905
*Punctelia jeckeri*	0	0	1	1	0	0,005	0,669	0,952
*Punctelia subrudecta*	0	0	1	1	0	0,004	0,666	0,952
*Apatococcus ammoniophilus*	0	0	0	1	1	0,004	0,706	0,952
*Dicranoweisia cirrata*	0	0	0	1	1	0,004	0,737	0,667
*Grimmia pulvinata*	0	0	0	1	1	0,015	0,813	0,474
*Orthotrichum diaphanum*	0	0	0	1	1	0,009	0,799	0,857
*Phaeophyscia orbicularis*	0	0	0	1	1	0,005	0,692	1
*Syntrichia papillosa*	0	0	0	1	1	0,033	0,614	0,905

**Fig. 6:**
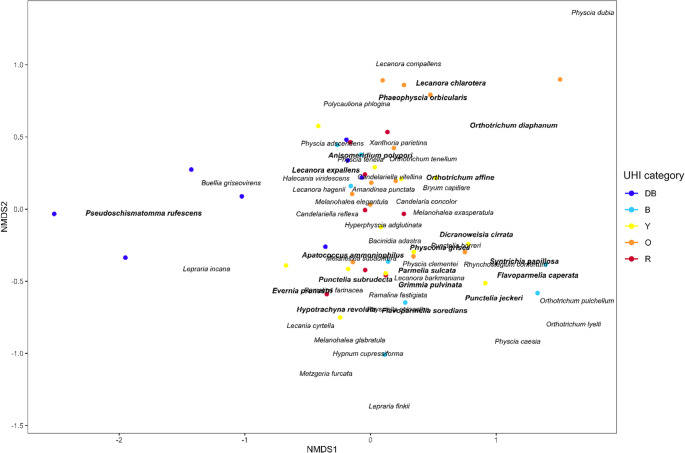
Non-metric multidimensional scaling (NMDS) plot for every sensor, coloured according to the urban heat island (UHI) zone in which it was situated (dark blue (“DB”): 0–0.5 °C; blue (“B”): 0.5–1.0 °C; yellow (“Y”): 1.0–1.5 °C; orange (“O”): 1.5–2.0 °C; red (“R”): > 2.0 °C). All species recorded on the sensor trees are plotted, with indicators in bold (19 out of the 22 indicator species listed in Table 2; the remaining three did not occur on the sensor trees). (Final stress = 0.09, stress-based R^2^ = 0.99, fit-based R^2^ = 0.92)

The results of the NMDS are visualised in Fig. 6 and Online Resource 9, where the sensors are plotted in relation to the species and environmental variables, respectively. From Fig. 6, we see that all indicators from zone “DB” and “B” are more situated on the left side of the NMDS, whereas most indicators from “B”, “Y” and “O” can be found on the bottom right, most indicators from “O” and “R” on the upper right and non-indicators (mostly indicators for nitrogen deposition) are scattered throughout the NMDS plot. In Online Resource 9, the NMDS with the environmental variables is given. The arrows of “RH_S” and “RH_W” point more to the left towards the sensor trees in the “DB” UHI zone. The other microclimatic variables (“T_S”, “T_W”, “VPD_S”, and “VPD_W”) are pointing more to the (bottom) right corner of the NMDS plot. Thus, we can infer that zones “DB” and “B” are characterized by increased RH in both winter and summer, and a lower VPD and temperature. Zones “B”, “Y”, and “O” are characterized by a mix of lower temperature, average RH in summer, but a lower RH in winter. Zones “O” and “R” have a higher temperature and VPD, but a lower RH. Non-indicators in the very centre of the NMDS plot are situated where there is no strong trend in RH, temperature, and VPD. Unexpectedly, we found that sensor trees from the UHI zones “B” to “R” did not show a clear correlation with the expected increase in temperature, RH and VPD (Online Resource 9).

## Discussion

To our knowledge, this is the first study that attempted to disentangle the effects of the urban environment on epiphytic lichens and bryophytes on a macro- and microscale, comparing data on city-scale temperature distributions with local-scale measurements. Timans et al. (2025) recently analysed distribution patterns of epiphytic lichens and bryophytes in Amsterdam, including a detailed analysis of trees in a single street, but without measuring local-scale climatic variables themselves. Although a lot of research has been conducted in urban environments, the main focus has so far been on air pollution resulting from excessive nitrogen and sulphur, or phorophyte characteristics such as bark pH. Research on other effects, such as climate change and, as an extension, the urban heat island effect (UHI), has increasingly gained attention over the years. However, data relating to the UHI is often retrieved from coarse-grained sources, proxies for the UHI characteristics (e.g., forest cover and vegetation structure), or fine-grained data from only a small number of meteorological stations. Here, we characterize the diversity of lichens and bryophytes on street trees in relation to the UHI and demonstrate that microclimatic variation is concordant with macroclimatic variation in defining species that can be used as UHI indicators.

### Urban epiphyte diversity and vegetation characterization

The present data confirm the conclusion by Timans et al. (2025) for Amsterdam that street trees harbour a considerable diversity of epiphytic species under the contemporary urban environmental conditions in western Netherlands. Furthermore, they confirm that *Tilia* trees are generally favourable phorophytes for epiphyte growth. Urban epiphytic lichens and bryophytes in the study area are generally xerophytic, photophilic, and mildly to very nitrophytic (Online Resource 2). This is concordant with the study by Timans et al. (2025), where species had generally low moisture values, high light values, intermediate eutrophic level values, and intermediate temperature values. The differences between the distribution of EIVs in both studies may result from different species compositions, considering that Timans et al. (2025) investigated eight different tree species in only Amsterdam, while also mapping the entire trunk between zero and two meters.

### Influence of environmental variables

We found that diversity and evenness for all epiphytes and lichens individually show an optimum at intermediate UHI values (unimodal peak), whereas bryophytes alone show a positive linear increase in biodiversity across the UHI gradient (Figs. 2 and 3; Table 1). Zeta diversity was structured along the UHI gradient (Online Resource 5); and species composition shifted along a gradient of the UHI (Fig. 4). Both the UHI and trunk diameter at breast height (DBH) were shown to significantly influence the biodiversity of lichens and bryophytes (Fig. 5; Table 1). Despite the result that bryophytes showed a more linearly increasing relationship with the UHI (Fig. 3B), we expect that bryophytes do show a unimodal relationship with the UHI, but with a peak in a higher UHI range. Indeed, personal observations in Madrid, a southern European city with a higher heat stress range, showed that the typical acrocarpous bryophytes are present in the suburbs instead of the city centre, where hardly any epiphytes are present. The bryophytes found in our dataset may also show higher resistance to pollution which could contribute to the observed positive linear pattern, although data to support this is lacking. Since most bryophyte indicators in the highest UHI zone show adaptations to increased temperature (hair-points and leaves curled when dry), temperatures seem to be the driving factor. We want to emphasize that the UHI map was derived from wind speed and population density data and recalibrated to account for soil sealing, vegetation cover, and land use (Remme et al. 2017). Since multiple factors are at play here, and despite that, it represents the UHI very well, it may also serve as a proxy for other urbanization factors. The unimodal or “suburban peak” pattern in lichens has been documented in other taxa, including birds (Crooks et al. 2004; Peng et al. 2024), amphibians (e.g., Yang et al. 2022), and plants (Ranta and Viljanen 2011; Wang et al. 2020; Zhang et al. 2024), particularly in temperate zones. Such patterns are often attributed to greater habitat heterogeneity in suburban environments, where mosaics of natural and modified habitats can support both urban-tolerant generalists and remnant specialists (e.g., McKinney 2008). While moderate urbanization may increase habitat heterogeneity due to the mixture of anthropogenic and semi-natural areas, higher levels of urban development generally reduce this heterogeneity again (McKinney 2006; Hou et al. 2023). Our findings align with these patterns, showing that biodiversity peaks at intermediate urbanization, but declines again in highly urbanized areas. Since we were unable to discern the influences of temperature and relative humidity (RH; Online Resource 9), our findings correlate with the UHI and UDI simultaneously.

Despite the species diversity peak at intermediate UHI values, all zones except “DB” exhibited a relatively flat zeta diversity decline curve and converging retention rates across zeta orders (Online Resource 5). This pattern indicates the presence of a consistent set of species shared across sites, reflecting biotic homogenization, likely driven by widespread, urban-tolerant generalists dominating these assemblages (McKinney 2006). This is consistent with the observed species assemblage of xerophytic, photophilic, and nitrophytic species. In contrast, the “DB” zone is characterized by both low biodiversity and low evenness (Fig. 3, Online Resource 4) as well as a steep zeta diversity decline and rapid drop in retention rates, suggesting species assemblages dominated by a few species and high species turnover between sites. We hypothesize that this pattern results from greater habitat heterogeneity within “DB” areas, where sites were located in ecologically diverse environments such as forests, forest edges, and open agricultural landscapes, which were largely absent from other zones. Such environmental heterogeneity can enhance species turnover by supporting distinct, habitat-specific species pools (e.g., more shade-adapted (sciophilous) species in forests or forest edges), contributing to the observed differences in zeta diversity and evenness patterns (Stein et al. 2014). Despite the borderline non-significant influence of light availability on the biodiversity, we cannot discern a potential confounding effect of streetlamps on the epiphytic community.

Despite the high prevalence of nitrophytic species, our results indicate that nitrogen did not prove to be a significant influence on the observed biodiversity pattern (Table 1). However, nitrogen pollution (in cities mainly NO_x_) is still an important factor at play regarding the community composition (as opposed to the biodiversity pattern) of urban lichens and bryophytes, because of multiple reasons. Firstly, when inspecting the employed NO_2_-data, we see high NO_2_-concentrations across each of the three cities. The gradient of NO_2_ is therefore small but not absent, as can be seen in the NMDS-results in Fig. 4. Here, NO_2_ is a significant factor explaining the species composition along the first NMDS-axis, where it distinguishes between sampling sites from the UHI zone “DB” and all the other UHI zones. Secondly, the high NO_2_-concentrations in the urban environment explain the high prevalence of nitrophytic species. Hence, we suggest that our findings related to the effect of the UHI on epiphytic biodiversity and species composition are especially applicable in urban environments with a low NO_2_-gradient. This is the case in cities with an overall high nitrogen load, as in our case, but also in cities with an overall low nitrogen load, as suggested by Munzi et al. (2014).

### Indicators for the UHI

In our study, 23 biological indicators were found that have the potential to measure the UHI. Stapper (2022) defined urbanotolerant and -phobic species using an indicator species analysis in Düsseldorf, Germany. Several consistent findings between both studies are visible such as *Apatococcus ammoniophilus*, *Phaeophyscia orbicularis* (Neck.) Moberg and *Orthotrichum diaphanum* Schrad. ex Brid. as urbanotolerant indicators and *Evernia prunastri* (L.) Ach., *Flavoparmelia caperata* (L.) Hale, *F. soredians* (Nyl.) Hale, *Hypotrachyna revoluta* and *Parmelia sulcata* Taylor (in our study indicator of “DB” and “B + Y”) as urbanophobic ones. *Dicranoweisia cirrata* (Hedw.) Lindb., *Lecanora expallens*,* Orthotrichum affine* Schrad. ex Brid., *Punctelia jeckeri* (Roum.) Kalb and *P. subrudecta* (Nyl.) Krøg were defined by Stapper (2022) as urbanophobic species, while our study shows that they are indicators of the intermediate UHI zones (“Y”, “O” and “R”). This may indicate that the distinction between urbanotolerant and urbanophobic species is based on other factors than the UHI as well.

Stapper (2022) defined the free-living algae of the genus *Trentepohlia*, the photobiont of the lichen indicators from the UHI zone “DB” in our study, as urbanophobic, which may explain why our indicators in this zone all share this characteristic. Indeed, it has been shown that crustose lichens and lichens with a *Trentepohlia* photobiont (traits which are present in our “DB” indicators) are associated to higher humidity (Matos et al. 2015) or a low urbanization (Koch et al. 2019). Other findings of the latter study also seem to hold up, namely that foliose, sorediate lichens are associated with medium to high urbanization. Additionally, the dominance of acrocarpous bryophytes as indicators for the highest UHI zones (“O” & “R”) holds up with the increased abundance of acrocarpous bryophytes during experimental warming in the alpine tundra (Alatalo et al. 2014) and their prevalence on sun-exposed concrete in Dutch cities compared to pleurocarpous bryophytes preferring shaded concrete (Veeger et al. 2025). Physiological evidence of the effect of the UHI on lichens is provided by Claerhout et al. (2025), showing that *Phaeophyscia orbicularis* and *Punctelia subrudecta* were found to have an increased specific thallus mass (dry weight per unit of area) in urban environments compared with their rural counterparts.

Many of our proposed lichen indicators, such as *Flavoparmelia caperata*,* F. soredians*,* Hypotrachyna revoluta*,* Punctelia jeckeri* and *P. subrudecta*, are already used as climate change indicators in Germany (Verein Deutscher Ingenieure 2017). Furthermore, all lichen indicators in our study were already reacting positively to climate change between 1979 and 2001 (van Herk et al. 2002), except for *Evernia prunastri*, which showed a negative trend, and *Pseudoschismatomma rufescens* (Pers.) Ertz & Tehler, which showed no trend. Furthermore, several indicators are also defined as indicators of nitrogen pollution, such as *Phaeophyscia orbicularis* and *Physconia grisea* (Lam.) Poelt (van Herk et al. 2003; Verein Deutscher Ingenieure 2023). Most suggested indicators have an EIV *N* > 5, and only *Lecanora expallens* and *Orthotrichum affine* have *N* > 3 (Online Resource 2). We may therefore conclude that indicators of the UHI are generally (modest) nitrophytic species with a varying tolerance to moisture and temperature.

Three species were expected as indicators for the UHI but were not specified as such by the indicator species analysis (ISA). The first is *Punctelia borreri* (Sm.) Krog as an indicator for intermediate UHI zones (“B”, “Y” & “O”), since it is a thermophilous, relatively common species in urban environments and has largely the same ecology as *P. jeckeri* and *P. subrudecta* (Timans et al. 2025). Furthermore, we expected *Ramalina fastigiata* (Pers.) Ach. and especially *R. farinacea* (L.) Ach. to be indicative of low UHI zones, because of its main distribution in the city outskirts, and since fruticose species like *Ramalina* spp. and *Evernia* spp. showed a negative trend with aridity as argued by Matos et al. (2015). Since these fruticose species have a typically low coverage in urban environments (pers. obs.), they often fell outside the ranges of the sampling ladder.

Based on the reported UHI indicators and biodiversity patterns, existing biomonitoring programs using epiphytic lichens and bryophytes, such as the nitrogen pollution monitoring program in the Netherlands since the 1980’s (van Herk et al. 2002; Aptroot and van Herk 2007; Sparrius 2007) or other international programs (see Counoy et al. 2025) can be expanded to heat/drought stress monitoring in urbanized areas. Such complementary monitoring programs are able to inform us about the extent of the UHI and take the effect of the UHI on the biodiversity of epiphytes into account when reporting their results (van der Kolk et al. 2024). Additionally, since the proposed UHI indicators have a broader geographic distribution, they can readily be applied in northwestern Europe within policy frameworks such as the Dutch National Climate Adaptation Strategy or the EU Urban Agenda.

### Microclimatic niche of UHI indicators

The proposed indicators were distributed along the gradient of microclimatic data (relative humidity (RH), temperature (T) and vapour-pressure deficit (VPD) in a manner that is concordant with the macroclimatic UHI data. As a result, we could calculate the microclimatic niche of these indicators in the investigated urban environment. This is a major advantage compared to assessing biodiversity or species composition data using broad-scale environmental variables related to air pollution or climate (including the UHI). These broad-scale variables are often the result of remote-sensing or statistical inference or interpolation, where observations from a relatively small number of data sources (e.g., meteorological stations) are used to calculate the environmental conditions in their vicinity. Although these have merits of their own, they do not always reflect the conditions as perceived by a species or community (e.g., Rutherford and Rebertus (2022) for lichens, Suren (1996) for bryophytes, Oda et al. (2019) for palms, Ficetola et al. (2018) for salamanders).

Species that are indicative of higher UHI zones experience a higher temperature in summer and winter, and a lower RH and higher VPD in summer only (Online Resource 7). Additionally, the range of these microclimatic variables all increased in summer, but not in winter (Online Resource 7). Therefore, it shows that species in high UHI zones have a broader niche in the urban environment and vice versa. This makes sense during summer, when the temperature differences of the UHI are highest. Namely, high UHI indicators are also present in non-urban environments, while those of low UHI zones are more restricted in their distribution due to environmental filtering. During winter, microclimatic data of the sensors show that with increasing UHI, the temperature and VPD rise, but not as steeply as in summer, while RH stays relatively the same. Furthermore, the temperature range decreases in winter, while the range of RH and VPD stays constant. As a result, the environmental conditions across the UHI gradient are more similar in winter than in summer, which explains the absence of an increasing range in winter for the microclimatic variables of the indicators.

Our indicators are also validated through the NMDS analysis in Fig. 6 and Online Resource 9. Indicators were generally sorted along the first NMDS axis. Unexpectedly, in Online Resource 9 A we can see that the sensors’ UHI zones do not correspond perfectly with the microclimatic variables. These variables make a clear distinction between sensors in zone “DB” and the other zones, but within the other zones, no real pattern emerges. This lack of trend in the NMDS graph may also be due to the relatively few sensor points (*n* = 12). However, the indicators did in fact correlate with both the broad-scale environment and the microclimate variables, and the summary statistics did show clear trends (Online Resource 7). Therefore, we infer that in our case, both scales are in fact concordant with each other in terms of species composition. Nonetheless, it is clear that the microclimate of urban roadside trees is more nuanced and complex than the broad-scale environmental variables suggest, and different urbanization-related variables may be more influential at the microscale (e.g., light from streetlamps). These findings have also been found in other research fields such as species distribution modelling (SDM; Haesen et al. 2023; König et al. 2024).

Although our research proved the importance of climatic variables in the distribution of lichens and bryophytes, we were not able to quantify the effect of nitrogen pollution due to the low gradient of pollution in our study area. We suggest future research to disentangle the effects of pollution and climate in cities with a more pronounced gradient of pollution, both across geographic areas and urban environments themselves.

## Conclusions

We showed how urban lichen and bryophyte diversity is affected by the urban heat island effect (UHI) and defined indicators that can be used to monitor the UHI in the future. The observed lichen and bryophyte biodiversity patterns are explained by the UHI and the age of trees. Lichen biodiversity follows a unimodal distribution along the UHI gradient, whereas the bryophytes exhibited a positive linear trend with increasing UHI. A small subset of common species is very dominant, and the overall species composition can be explained by the gradient in the UHI and nitrogen dioxide (NO_2_) concentration. A total of 23 indicators (including species combinations, one alga and the total species coverage) were identified for five different UHI zones. Finally, the sensor data (temperature, relative humidity and vapour-pressure deficit) measuring the microclimate confirmed our findings. We expect that our results will form a baseline for understanding future changes in diversity and community composition of epiphytic lichens and bryophytes in the urban environment, both in light of an increasingly changing climate, which reinforces the UHI, but also in light of urban greening measures to counteract this. Furthermore, the results provide a foundation for scientists, municipalities, and urban planners to use the indicators to map the UHI or assess mitigation actions using biomonitoring or to apply the findings in ongoing monitoring schemes.

## Supplementary Information

Below is the link to the electronic supplementary material.

Supplementary Material 1 (XLSX 603 KB)

Supplementary Material 2 (PDF 368 KB)

Supplementary Material 3 (PDF 1.00 MB)

Supplementary Material 4 (R 28.8 KB)

Supplementary Material 5 (XLSX 65.6 KB)

Supplementary Material 6 (XLSX 70.0 KB)

Supplementary Material 7 (PDF 335 KB)

Supplementary Material 8 (PDF 381 KB)

Supplementary Material 9 (XLSX 21.1 MB)

## Data Availability

The authors confirm that the data supporting the findings of this study are available within the article and its Online Resources.
