# Supplementary material for: Urban heat Islands shape epiphytic communities of lichens and bryophytes
Source: Urban Ecosyst. 2026 Feb 21;29(2):63. doi: 10.1007/s11252-026-01930-8 (PMC12923442; doi:10.1007/s11252-026-01930-8)
Supplement: Supplementary file 2 — Supplementary Material 2 (PDF 368 KB) [file 11252_2026_1930_MOESM2_ESM.pdf]

Supplementary Material 2 – Observed species and Ellenberg values

Article title: Urban heat island shapes epiphytic communities of lichens and bryophytes
Journal name: Urban Ecosystems
Author names and affiliation:

- Tim Claerhout: Naturalis Biodiversity Center, Leiden, The Netherlands; Hortus botanicus Leiden, Leiden University, Leiden, The Netherlands; Institute of Biology Leiden, Leiden University, Leiden, The Netherlands
- Laurens B Sparrius: BLWG, Utrecht, The Netherlands
- Paul JA Keßler: Hortus botanicus Leiden, Leiden University, Leiden, The Netherlands; Institute of Biology Leiden, Leiden University, Leiden, The Netherlands
- Michael Stech: Naturalis Biodiversity Center, Leiden, The Netherlands; Leiden University, Leiden, The Netherlands.

E-mail address of corresponding author: t.claerhout@hortus.leidenuniv.nl

Caption: List of observed species, their total abundance and Ellenberg value (F, L, R, N, T & K)

Lichen Ellenberg-data: Sparrius, L.B., A. Aptroot & C.M. van Herk (2015) Ecological indicator values of lichens in the Netherlands. BLWG.
Bryophyte Ellenberg data: Siebel, H. (2005) Indicatiewaarden van mossen [Indicator values of bryophytes]. BLWG.

Tables

Table 1 Species list with their abundance (summed averaged abundance across all sampling sites) , amount of presences in the number of squares of the sampling ladder (**#Sq**), amount of presences on sampled trees (**#Tr**), amount of presence in the number of sampling sites (**#SS**), Ellenberg values

Species	Abundance	#Sq	#Tr	#SS	F	L	R	N	T	K
Alyxoria varia	0,33	1	1	1	3	7	5	5	5	3
Amandinea punctata	1151,40	1783	233	94	1	9	5	7	5	5
Anisomeridium polypori	88,40	86	17	11	5	3	5	5	5	3
Arthonia radiata	42,07	88	27	19	1	9	5	5	5	4
Arthonia spadicea	188,67	82	11	9	3	3	3	3	5	5
Athallia cerinella	0,07	1	1	1	1	9	5	7	5	3
Bacidina adastra	84,07	166	52	37	3	7	5	7	6	3
Brachythecium rutabulum	1,27	5	5	5	5	4		8		5
Bryum argenteum	4,33	22	14	12		7	7	8	5	
Bryum capillare	40,33	100	36	25	5		7	5		5
Bryum dichotomum	0,27	4	1	1	6	8	7	8	6	5

Buellia griseovirens	4,53	15	11	11	3	7	5	5	5	5
Candelaria concolor	2171,60	3599	258	94	3	9	5	9	5	3
Candelariella reflexa	367,87	878	122	67	3	9	5	9	5	4
Candelariella vitellina	63,80	131	38	29	1	9	3	7	5	5
Candelariella xanthostigma	2,80	17	10	10	3	9	5	7	5	5
Catillaria nigroclavata	19,53	68	21	17	1	9	5	9	5	5
Chaenotheca trichialis	29,07	11	2	2	3	5	3	1	5	5
Cladonia fimbriata	8,67	14	5	4	3	9	5	5	5	5
Coenogonium pineti	0,07	1	1	1	3	3	3	5	5	5
Dicranoweisia cirrata	46,33	168	55	34	4	7	5	4	5	4
Diploicia canescens	0,47	2	2	2	1	9	7	7	7	4
Evernia prunastri	56,20	101	47	35	3	9	3	3	5	5
Flavoparmelia caperata	163,47	154	44	30	3	9	5	5	7	4
Flavoparmelia soredians	245,87	365	101	58	3	9	5	5	8	3
Frullania dilatata	16,20	27	18	17	4	6	6	3	3	5
Graphis scripta	0,33	3	3	3	3	3	3	3	5	5
Grimmia pulvinata	12,73	51	30	23	2	8	8	3	5	5
Halecania viridescens	17,47	66	21	17	3	7	5	7	7	3
Homalothecium sericeum	7,67	9	5	5	2	7	7	4	3	5
Hyperphyscia adglutinata	2031,13	2450	266	99	1	9	5	9	7	3
Hypnum andoi	2,67	2	1	1	5	3	3	2	4	3
Hypnum cupressiforme	456,67	274	70	43	4			3		5
Hypogymnia physodes	6,53	17	12	9	3	9	1	1	5	5
Hypogymnia tubulosa	5,33	9	5	5	3	9	3	3	5	5
Hypotrachyna revoluta	226,07	244	75	48	3	7	3	5	7	3
Lecania cyrtella	0,67	8	8	8	3	7	5	7	5	5
Lecanora barkmaniana	19,87	65	32	25	3	9	5	7	8	3
Lecanora carpinea	13,60	33	18	15	3	9	5	5	5	5
Lecanora chlarotera	18,93	72	46	34	3	9	5	5	5	5
Lecanora compallens	30,13	132	46	37	3	9	3	3	6	3
Lecanora conizaeoides	0,33	1	1	1	3	9	1	1	5	5
Lecanora dispersa	0,80	5	4	4	3	9	9	9	5	5
Lecanora expallens	489,93	1023	196	88	3	7	3	3	5	5
Lecanora hagenii	0,27	2	2	2	1	9	5	9	5	5
Lecanora horiza	0,67	6	5	5	3	9	5	5	7	2
Lecanora muralis	0,07	1	1	1	3	9	7	9	5	5
Lecanora saligna	0,07	1	1	1	3	9	3	5	5	5
Lecidella elaeochroma	275,33	509	105	54	1	9	5	5	5	5
Lepraria finkii	43,93	58	19	14	5	5	5	3	5	5
Lepraria incana	287,53	500	78	41	3	5	3	1	5	5
Lepraria rigidula	0,07	1	1	1	3	7	3	5	5	5
Melanelixia subaurifera	228,13	45	9	6	3	9	5	5	5	5

Melanohalea elegantula	12,93	462	120	63	3	7	5	5	6	4
Melanohalea exasperatula	20,13	29	8	6	3	7	5	5	5	5
Melanelixia glabratula	38,80	24	7	6						
Metzgeria furcata	27,53	14	5	5	4	4	6	4	3	5
Naetrocymbe punctiformis	5,33	2	1	1	1	9	5	5	5	4
Normandina pulchella	6,73	11	6	6	3	7	5	5	5	2
Opegrapha niveoatra	6,73	12	5	5	3	7	5	5	5	3
Orthotrichum acuminatum	1,07	3	3	3	3	6	6	4	8	6
Orthotrichum affine	154,53	300	84	48	4	6	6	3	4	5
Orthotrichum anomalum	5,53	20	9	8	2	8	7	3	3	5
Orthotrichum cupulatum	0,47	1	1	1	2	8	8	4	3	5
Orthotrichum diaphanum	185,47	435	94	57	2	8	7	7	6	5
Orthotrichum lyellii	15,60	21	9	9	4	6	5	3	4	4
Orthotrichum pulchellum	5,87	14	6	5	4	5	6	5	6	3
Orthotrichum striatum	3,13	12	9	7	5	6	7	3	5	4
Orthotrichum tenellum	1,93	7	5	5	3	7	7	5	6	4
Parmelia sulcata	431,13	526	133	67	3	7	5	5	5	5
Parmelina tiliacea	1,67	2	2	2	3	9	5	5	5	4
Parmotrema perlatum	18,67	35	20	16	3	7	5	5	7	4
Pertusaria pertusa	0,07	1	1	1	3	7	5	3	5	5
Phaeophyscia nigricans	11,47	39	19	19	3	9	7	9	5	5
Phaeophyscia orbicularis	1063,20	1555	214	90	3	9	7	9	5	5
Physcia adscendens	3068,87	3934	285	98	1	9	5	9	5	5
Physcia aipolia	0,67	4	4	4	3	9	5	5	5	5
Physcia caesia	64,60	108	39	28	1	9	7	9	5	5
Physcia clementei	2,13	4	4	4	3	9	5	5	8	3
Physcia dubia	19,07	33	15	12	3	9	7	9	5	5
Physcia tenella	1413,47	1481	163	70	3	9	5	7	5	5
Physcia tribacioides	1,20	3	3	2	3	9	5	7	7	4
Physciella chloantha	10,67	20	9	8	1	9	5	9	7	6
Physconia grisea	350,40	517	126	65	3	9	7	7	7	4
Pleurosticta acetabulum	4,67	1	1	1	3	9	5	5	7	5
Polycauliona candelaria	0,40	2	1	1	3	9	5	7	5	5
Polycauliona phlogina	0,20	1	1	1						
Polycauliona polycarpa	1,93	16	11	11	3	9	5	7	5	5
Porina aenea	0,40	2	1	1	3	1	5	5	5	5
Pseudoschismatomma rufescens	155,40	125	20	14	3	7	5	5	5	4
Punctelia borreri	221,20	271	80	55	3	9	5	5	8	3
Punctelia jeckeri	764,93	689	136	69	3	9	5	5	6	3
Punctelia subrudecta	434,33	873	168	78	3	9	5	5	7	4
Radula complanata	4,07	4	4	4	5	4	6	4	3	5

Ramalina farinacea	13,07	26	19	16	3	9	5	5	5	5
Ramalina fastigiata	3,80	8	6	4	3	9	5	5	5	5
Ramalina lacera	0,33	1	1	1	3	9	5	5	8	3
Rhynchostegium confertum	18,27	20	10	9	5	4	6	6	5	4
Rinodina oleae	0,27	3	2	2	3	9	7	9	5	5
Strangospora pinicola	0,80	6	3	2	3	9	3	5	5	5
Syntrichia laevipila	0,80	2	2	2	3	7	7	6	5	4
Syntrichia papillosa	174,60	380	83	46	3	6	7	6	6	4
Tortula muralis	1,87	11	8	7	3		8	6	5	5
Ulota bruchii	2,40	4	4	4	5	4	4	3	3	4
Ulota crispa	0,33	2	1	1	6	4	4	3	3	5
Xanthoria calcicola	2,60	5	4	4	3	9	7	7	7	2
Xanthoria parietina	737,40	1911	254	94	1	9	5	9	5	5
Apatococcus ammoniophilus	867,00	880	128	60						
Melanohalea/Melanelixia sp.	0,13	1	1	1						
Orthotrichum sp.	63,80	224	71	48						
Pertusaria sp.	0,07	1	1	1						
Ulota sp.	3,60	8	6	6						

Table 2 Frequency table of every Ellenberg class

Classes	*F*	*L*	*R*	*N*	*T*	*K*
0	0	0	0	0	0	0
1	14	1	2	4	0	0
2	5	0	0	1	0	3
3	67	5	15	17	8	18
4	7	6	2	6	3	20
5	9	4	54	39	63	61
6	2	6	7	4	9	2
7	0	21	19	16	13	0
8	0	5	3	3	6	0
9	0	54	1	15	0	0

Graphs

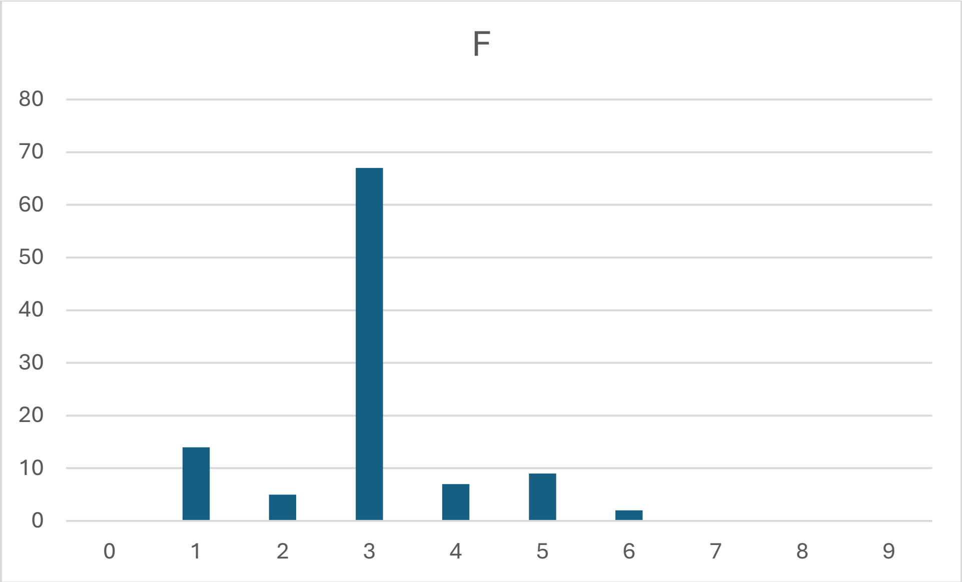

Fig. 1 Bargraph showing the spread of the soil moisture indicator value (F)

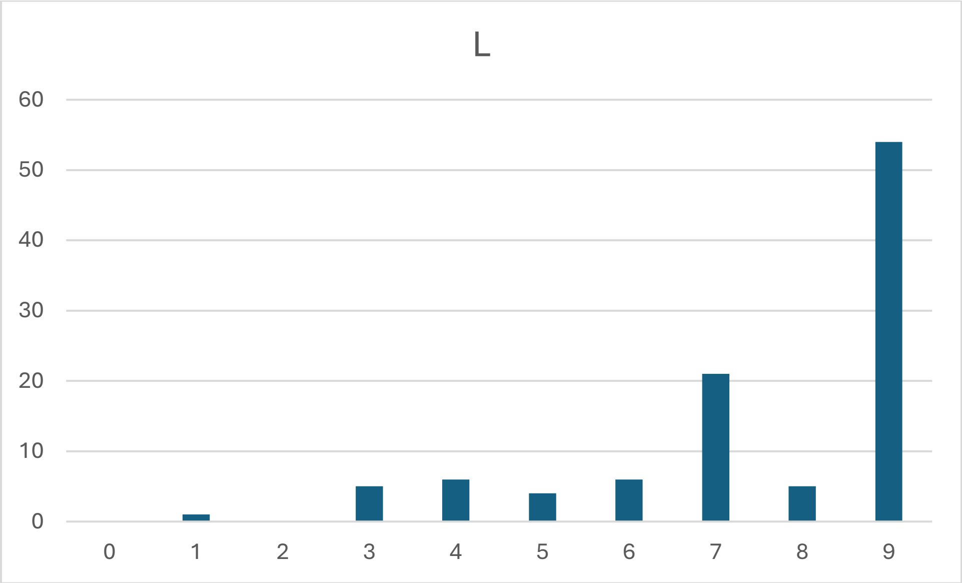

Fig. 2 Bargraph showing the spread of the light indicator value (L)

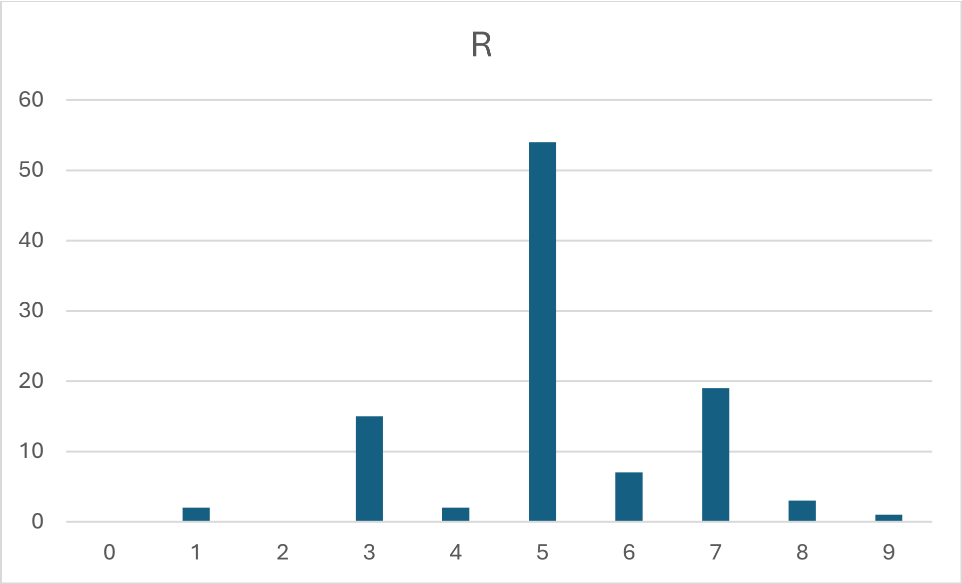

Fig. 3 Bargraph showing the spread of the soil reaction indicator value (R)

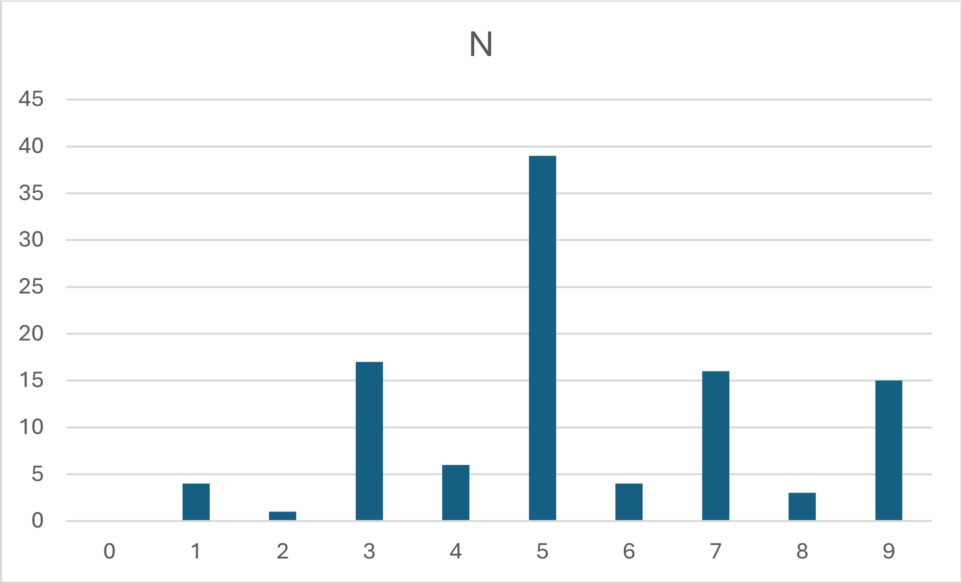

Fig. 4 Bargraph showing the spread of the nutrient availability indicator value (N)

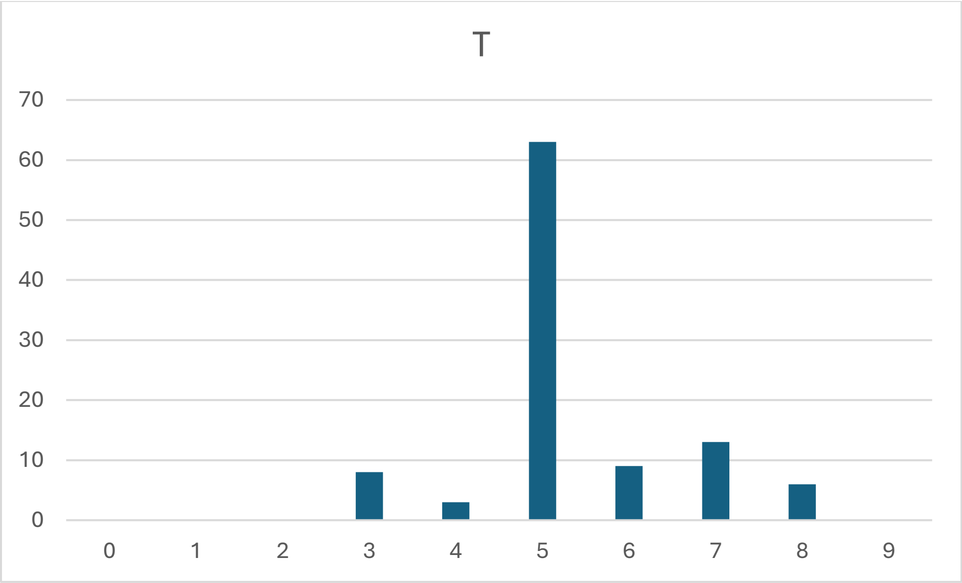

Fig. 5 Bargraph showing the spread of the temperature indicator value (T)

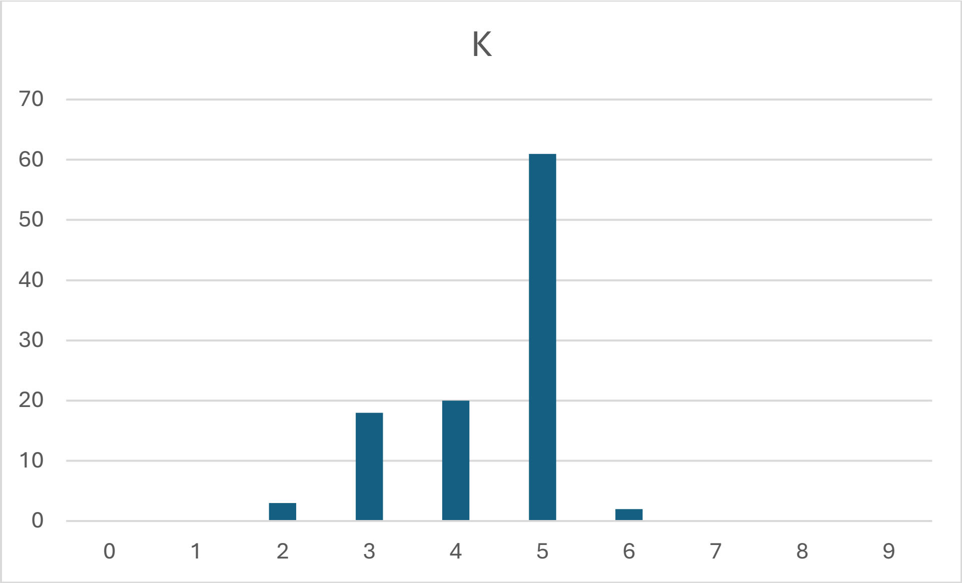

Fig. 6 Bargraph showing the spread of the continentality indicator value (K)
