# Supplementary material for: Urban heat Islands shape epiphytic communities of lichens and bryophytes
Source: Urban Ecosyst. 2026 Feb 21;29(2):63. doi: 10.1007/s11252-026-01930-8 (PMC12923442; doi:10.1007/s11252-026-01930-8)
Supplement: Supplementary file 3 — Supplementary Material 3 (PDF 1.00 MB) [file 11252_2026_1930_MOESM3_ESM.pdf]

Supplementary Data 3 – Bargraphs of abundance of species

Article title: Urban heat island shapes epiphytic communities of lichens and bryophytes
Journal name: Urban Ecosystems
Author names and affiliation:

- Tim Claerhout: Naturalis Biodiversity Center, Leiden, The Netherlands; Hortus botanicus Leiden, Leiden University, Leiden, The Netherlands; Institute of Biology Leiden, Leiden University, Leiden, The Netherlands
- Laurens B Sparrius: BLWG, Utrecht, The Netherlands
- Paul JA Keßler: Hortus botanicus Leiden, Leiden University, Leiden, The Netherlands; Institute of Biology Leiden, Leiden University, Leiden, The Netherlands
- Michael Stech: Naturalis Biodiversity Center, Leiden, The Netherlands; Leiden University, Leiden, The Netherlands.

E-mail address of corresponding author: t.claerhout@hortus.leidenuniv.nl

Caption: Bargraphs of the abundance of the top 10 species, top 25 species and all species, respectively. The total abundance reflects the summed average abundances across every sampling site

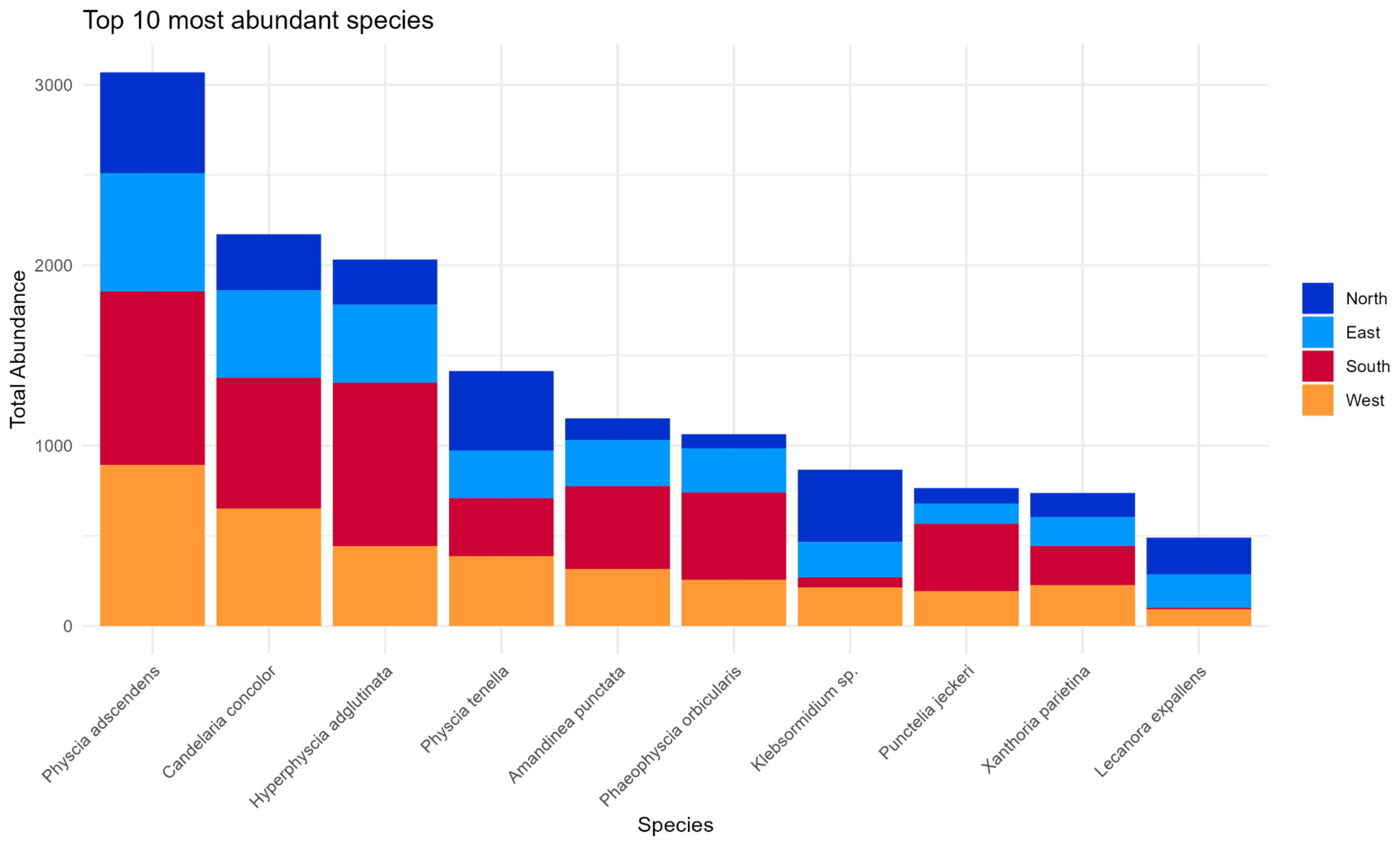

Fig. 1 Bargraph of the top 10 species, subdivided for every cardinal direction. Note that *Klebsormidium* sp. is now denoted as *Apatococcus ammoniophilus*

Classified as Internal | Intern

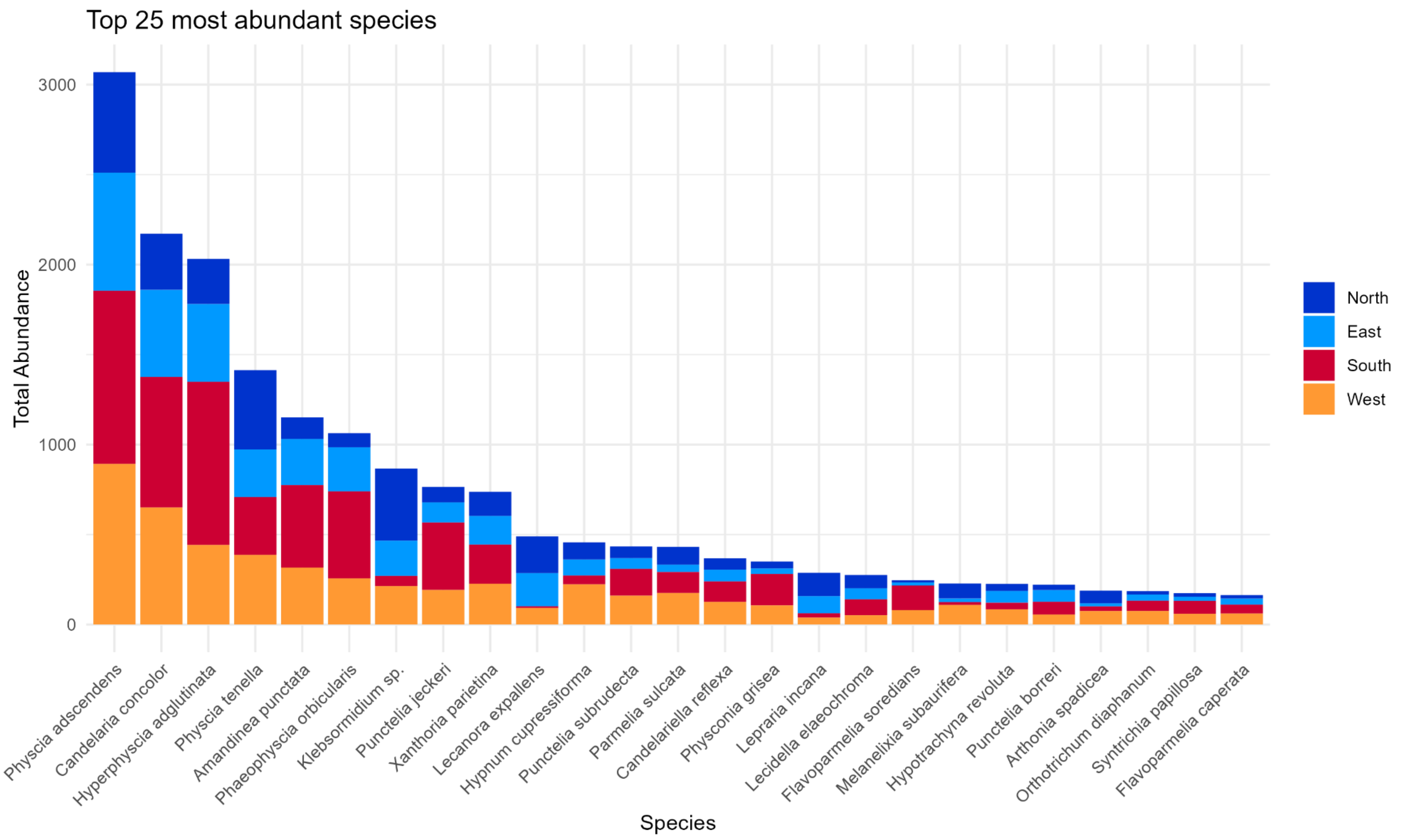

Fig. 2 Bargraph of the top 25 species, subdivided for every cardinal direction. Note that *Klebsormidium* sp. is now denoted as *Apatococcus ammoniophilus*

Classified as Internal | Intern

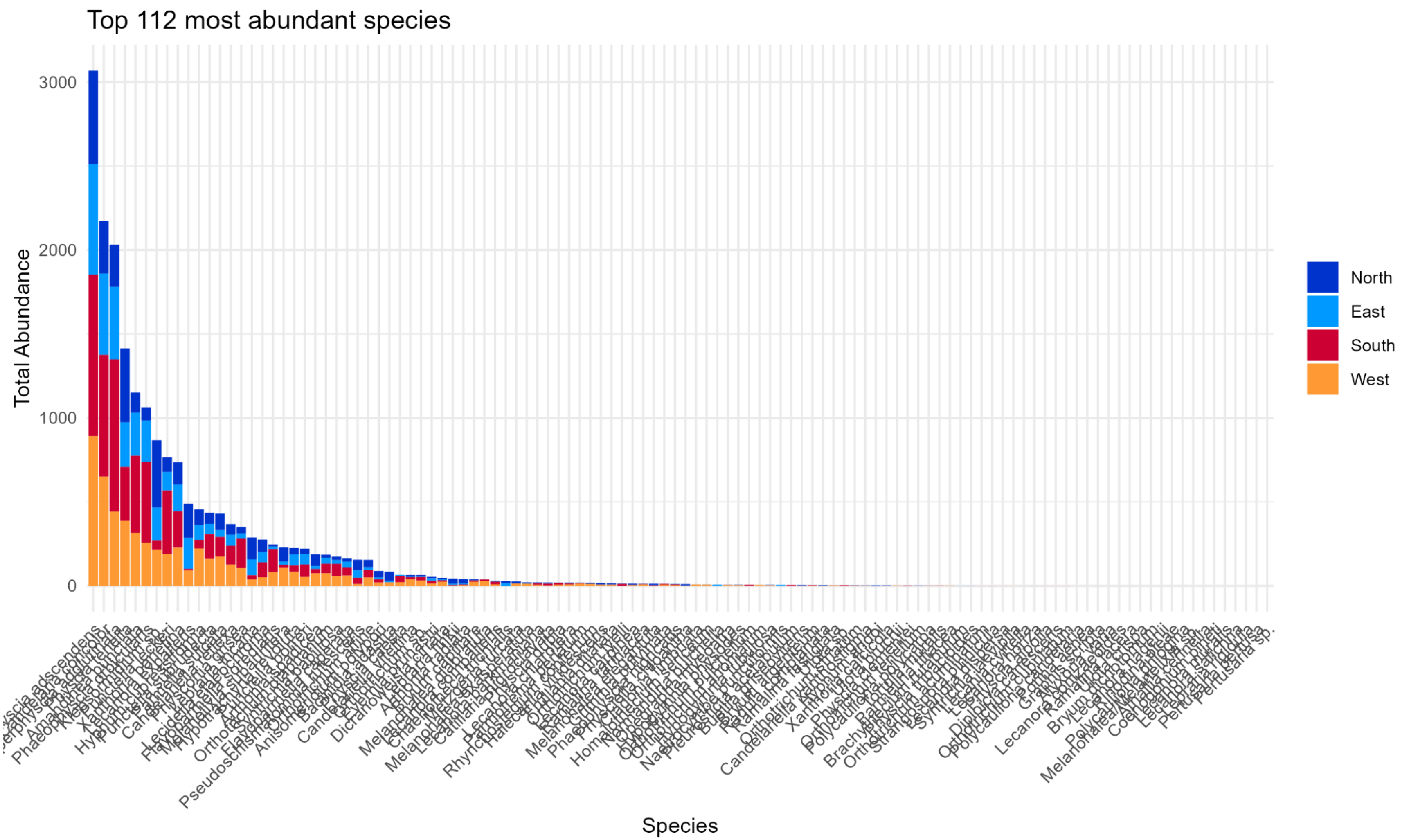

Fig. 3 Bargraph of all species found, subdivided for every cardinal direction. Note that *Klebsormidium* sp. is now denoted as *Apatococcus ammoniophilus*
