# Supplementary material for: Urban heat Islands shape epiphytic communities of lichens and bryophytes
Source: Urban Ecosyst. 2026 Feb 21;29(2):63. doi: 10.1007/s11252-026-01930-8 (PMC12923442; doi:10.1007/s11252-026-01930-8)
Supplement: Supplementary file 7 — Supplementary Material 7 (PDF 335 KB) [file 11252_2026_1930_MOESM7_ESM.pdf]

# Supplementary Material 5 – Zeta diversity decline

Article title: Urban heat island shapes epiphytic communities of lichens and bryophytes
Journal name: Urban Ecosystems
Author names and affiliation:

- Tim Claerhout: Naturalis Biodiversity Center, Leiden, The Netherlands; Hortus botanicus Leiden, Leiden University, Leiden, The Netherlands; Institute of Biology Leiden, Leiden University, Leiden, The Netherlands
- Laurens B Sparrius: BLWG, Utrecht, The Netherlands
- Paul JA Keßler: Hortus botanicus Leiden, Leiden University, Leiden, The Netherlands; Institute of Biology Leiden, Leiden University, Leiden, The Netherlands
- Michael Stech: Naturalis Biodiversity Center, Leiden, The Netherlands; Leiden University, Leiden, The Netherlands.

E-mail address of corresponding author: t.claerhout@hortus.leidenuniv.nl

Caption: Normalized Sørensen-equivalent zeta diversity decline for every urban heat island zone and the entire dataset, respectively

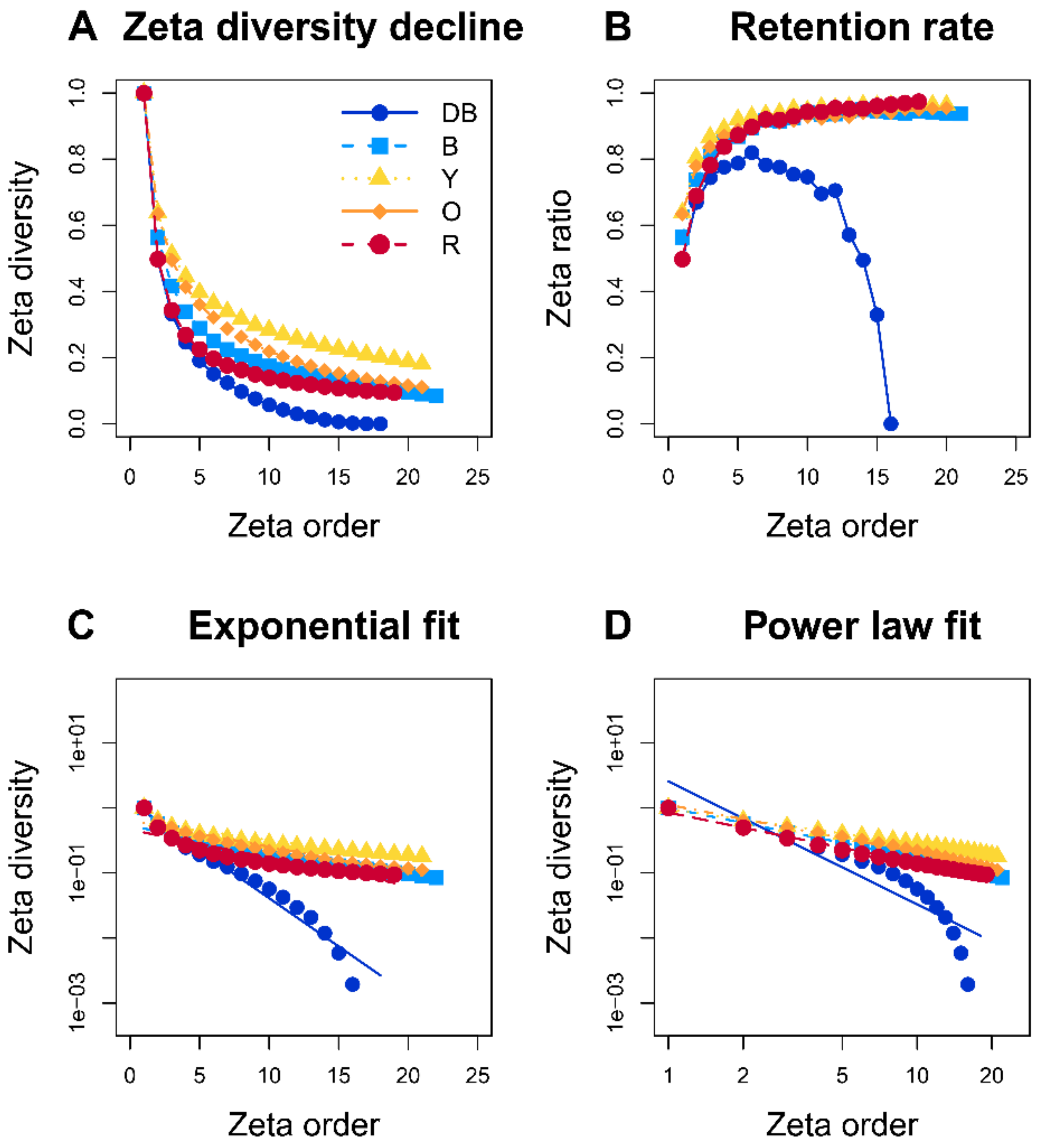


**Fig. 1** Normalized Sørensen-equivalent zeta diversity decline for every urban heat island zones (dark blue ("DB"): 0 – 0.5 °C; blue ("B"): 0.5 – 1.0 °C; yellow ("Y"): 1.0 – 1.5 °C; orange ("O"): 1.5 – 2.0 °C; red ("R"): > 2.0 °C). A. Decline of the number of shared species with increasing zeta order. B. Species retention rate, indicating "the degree to which common species are more likely to be retained in additional sites or samples than rare ones with an increase in zeta order" (McGeoch et al. 2019). C & D. Shape of zeta diversity decline compared with an exponential and power-law fit, respectively. The former indicates equal probability of species across sites, while the latter indicates unequal probabilities, implying the data is nonrandomly structured. The AIC values of the exponential and power-law fit are -8.72, -35.8, -46.1, -43.8 and -20.3, and 14.1, -116.8, -132.8, -94.5 and -75.6 for UHI zones "DB", "B", "Y", "O" and "R", respectively

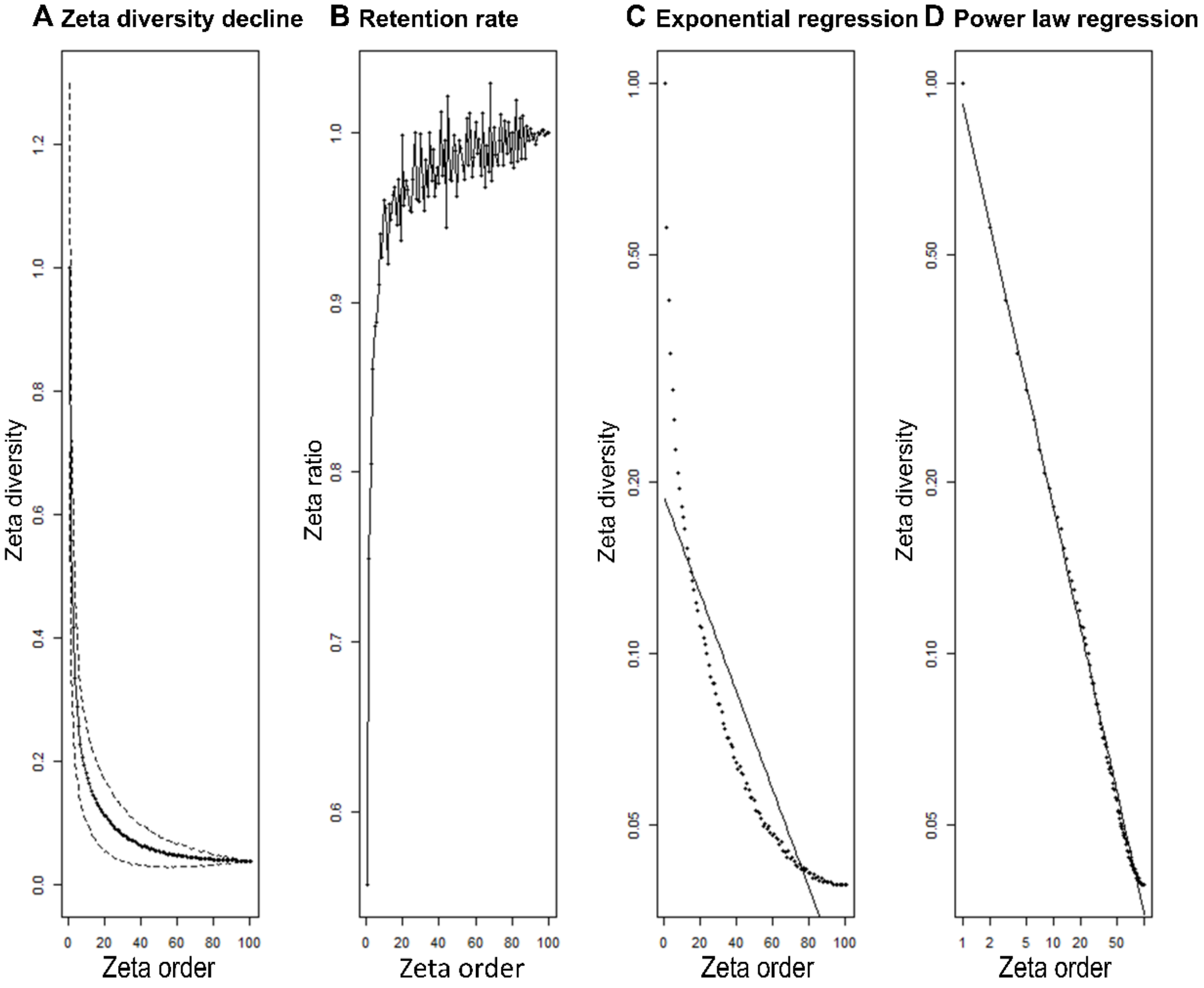


**Fig. 2** Normalized Sørensen-equivalent zeta diversity decline for the entire dataset. A. The decline of the number of shared species with increasing zeta order. B. The species retention rate, indicating “the degree to which common species are more likely to be retained in additional sites or samples than rare ones with an increase in zeta order” (McGeoch et al. 2019). C & D. The shape of zeta diversity decline compared with an exponential (AIC = 165.5- and power-law fit (AIC = 37.9), respectively. The former indicates equal probability of species across sites, while the latter indicates unequal probabilities, implying the data is nonrandomly structured
