# Supplementary material for: Urban heat Islands shape epiphytic communities of lichens and bryophytes
Source: Urban Ecosyst. 2026 Feb 21;29(2):63. doi: 10.1007/s11252-026-01930-8 (PMC12923442; doi:10.1007/s11252-026-01930-8)
Supplement: Supplementary file 8 — Supplementary Material 8 (PDF 381 KB) [file 11252_2026_1930_MOESM8_ESM.pdf]

Supplementary Data 9 – NMDS graphs with environmental fit and species fit

Article title: Urban heat island shapes epiphytic communities of lichens and bryophytes
Journal name: Urban Ecosystems
Author names and affiliation:

- Tim Claerhout: Naturalis Biodiversity Center, Leiden, The Netherlands; Hortus botanicus Leiden, Leiden University, Leiden, The Netherlands; Institute of Biology Leiden, Leiden University, Leiden, The Netherlands
- Laurens B Sparrius: BLWG, Utrecht, The Netherlands
- Paul JA Keßler: Hortus botanicus Leiden, Leiden University, Leiden, The Netherlands; Institute of Biology Leiden, Leiden University, Leiden, The Netherlands
- Michael Stech: Naturalis Biodiversity Center, Leiden, The Netherlands; Leiden University, Leiden, The Netherlands.

E-mail address of corresponding author: t.claerhout@hortus.leidenuniv.nl

Caption: Non-metric multidimensional scaling (NMDS) plots for every sensor, coloured according to the urban heat island (UHI) zone in which it was situated (dark blue (“DB”): 0 – 0.5 °C; blue (“B”): 0.5 – 1.0 °C; yellow (“Y”): 1.0 – 1.5 °C; orange (“O”): 1.5 – 2.0 °C; red (“R”): > 2.0 °C). Sensor names are derived from their number from Online Resource 7 with an extra suffix, indicating its cardinal direction (north (“_n”) or south (“_s”)). **A.** Environmental variables plotted as arrows indicating the strength of the environmental gradient for the variables “RH” (relative humidity; summer: $R^2 = 0.33$, $p < 0.01$; winter: $R^2 = 0.25$, $p < 0.01$), “T” (temperature; summer: $R^2 = 0.16$, $p < 0.05$; winter: $R^2 = 0.28$, $p < 0.01$) and “VPD” (vapour-pressure deficit; summer: $R^2 = 0.32$, $p < 0.01$; winter: $R^2 = 0.34$, $p < 0.01$). All variables are the mean value. The suffixes indicate the season for which the data was collected (“_S” for summer and “_W” for winter). **B.** Same graph as A., but with species from the raw vegetation data of the sensor trees plotted. Indicators are in bold. (Final stress = 0.09, stress-based $R^2 = 0.99$, fit-based $R^2 = 0.92$)

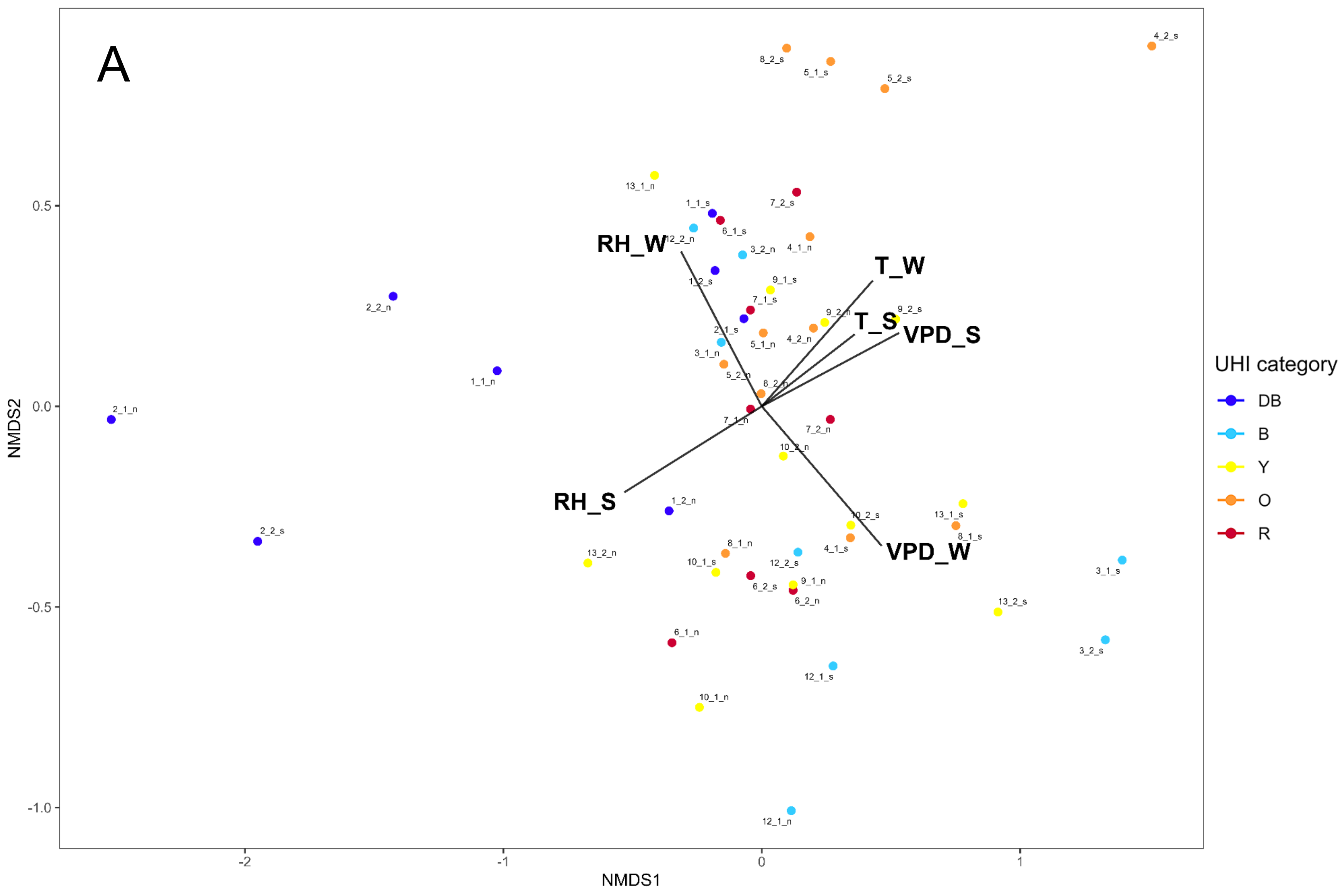
A
RH_W
T_W
T_S
VPD_S
RH_S
VPD_W
NMDS1
NMDS2
UHI category
DB
B
Y
O
R

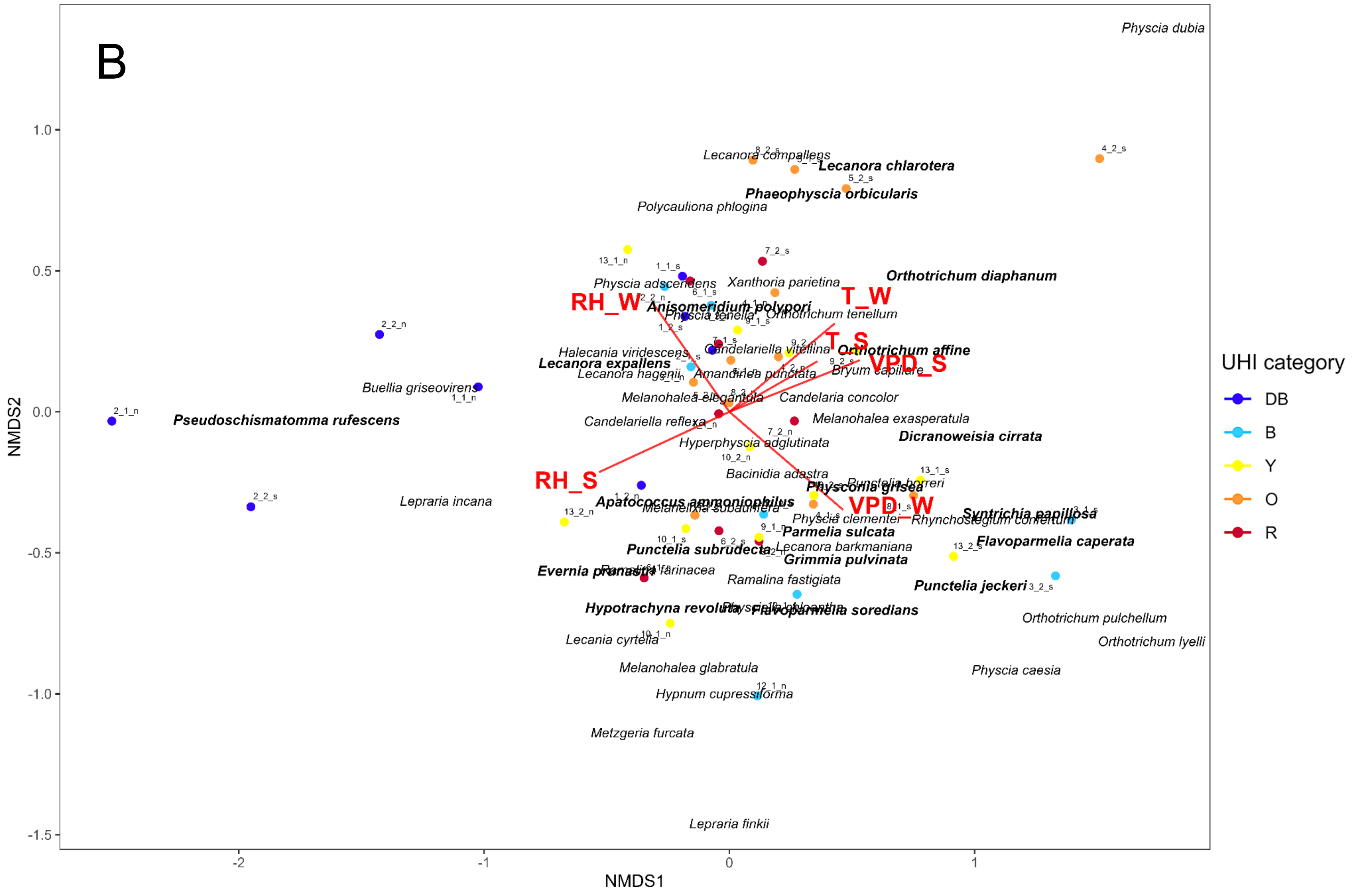

B
NMDS1
NMDS2
1.0
0.5
0.0
-0.5
-1.0
-1.5
-2
-1
0
1
UHI category
DB
B
Y
O
R
RH_W
T_W
T_S
VPD_S
RH_S
VPD_W
Physcia dubia
Lecanora chlarotera
Phaeophyscia orbicularis
Polycauliona phlogina
Orthotrichum diaphanum
Xanthoria parietina
Anisomeridium polypori
Orthotrichum tenellum
Halecania viridescens
Orthotrichum affine
Lecanora expallens
Bryum capillare
Buellia griseovirens
Candelaria concolor
Pseudoschismatomma rufescens
Candelariella reflexa
Melanohalea exasperatula
Dicranoweisia cirrata
Hyperphyscia adglutinata
Physconia grisea
Lepraria incana
Syntrichia papillosa
Parmelia sulcata
Flavoparmelia caperata
Punctelia subrudecta
Lecanora barkmaniana
Grimmia pulvinata
Ramalina fastigiata
Punctelia jeckeri
Flavoparmelia soredians
Orthotrichum pulchellum
Orthotrichum lyelli
Lecania cyrtella
Physcia caesia
Melanohalea glabratula
Metzgeria furcata
Lepraria finkii
